# Therapeutic Resistance in Melanoma: Molecular Mechanisms and Emerging Pharmaceutical Strategies

**DOI:** 10.3390/ph19091455

**Published:** 2026-09-14

**Authors:** Nicolas Moussallem, Ali Awada, Roy El Darzi, Ali Tarhini, Akel Khaled, Christopher Ashy, Wajih Nasr, Amal El Masri, George Saad, Mohamad Itani, Joe Rizkallah, Nicole Charbel, Zuhair Hatahet, Dana Saade, Jihane Abou Rahal, Firas Kreidieh

**Affiliations:** 1 School of Medicine, Case Western Reserve University, Cleveland, OH 44106, USA; 2 Division of Hematology and Oncology, Department of Internal Medicine, American University of Beirut, Beirut P.O. Box 11-0236, Lebanon; 3 Department of Diagnostic Radiology, American University of Beirut, Beirut P.O. Box 11-0236, Lebanon; 4 Department of Dermatology, American University of Beirut, Beirut P.O. Box 11-0236, Lebanon

**Keywords:** melanoma, therapeutic resistance, targeted therapy, immune checkpoint inhibitors, MAPK signaling, tumor microenvironment, phenotypic plasticity, drug development

## Abstract

Cutaneous melanoma remains a global health challenge. Although BRAF/MEK-targeted therapies and immune checkpoint inhibitors (ICIs) have improved outcomes in advanced disease, durable responses remain difficult to achieve for most patients. Therapeutic resistance represents the central barrier to long-term disease control. This review comprehensively examines the molecular and immunologic mechanisms underlying melanoma resistance and integrates these insights with emerging pharmaceutical strategies designed to overcome them. We explore the genetic landscape of melanoma, including oncogenic alterations in BRAF, NRAS, NF1, CDKN2A, and PTEN, and explain how dysregulation of the MAPK and PI3K/AKT/mTOR signaling axes drives therapeutic escape. Phenotypic plasticity is discussed as a critical epigenetic driver of drug tolerance. The tumor microenvironment (TME) is examined as an active co-conspirator in resistance, encompassing immunosuppressive cell populations, cancer-associated fibroblasts, and metabolic competition. Resistance mechanisms to targeted therapy, including MAPK reactivation, bypass signaling, transcriptional reprogramming, and metabolic rewiring, are reviewed alongside tumor-intrinsic and tumor-extrinsic mechanisms of ICI resistance. Emerging therapeutic strategies are surveyed, including next-generation RAF and ERK inhibitors, dual-pathway blockade, and metabolic therapies targeting oxidative phosphorylation. Innovations in molecular imaging, liquid biopsy, and artificial intelligence-driven biomarker discovery are highlighted as pivotal tools for real-time resistance monitoring and adaptive treatment. By linking mechanistic insights with translational advances, this review advocates for combination strategies and adaptive clinical frameworks to achieve more durable disease control in melanoma.

## 1. Introduction and Methodology

Cutaneous melanoma is the deadliest form of skin cancer and remains a growing global health challenge despite major therapeutic advances. In 2020, approximately 325,000 new melanoma cases and 57,000 melanoma-related deaths were reported, accounting for roughly 1.7% of all cancer diagnoses globally [1,2]. According to more recent statistics, over 100,000 cases of melanoma were diagnosed in 2025 in the United States alone [3]. These numbers illustrate the growing relevance of melanoma and highlight it as a notable contributor to the overall cancer burden. Importantly, melanoma incidence varies by region: the highest rates occur in Australia and New Zealand, followed by Western Europe and North America, whereas much lower rates are observed in Asia and Africa [4,5]. These differences likely reflect variability in ultraviolet radiation exposure, skin phototype distribution, and screening practices. Of even greater concern is the rising trajectory in melanoma incidence and clinical impact. If current trends persist, melanoma is expected to account for more than half a million new cases per year by 2040 [1]. Collectively, these epidemiological patterns underscore the clinical urgency for advances in both prevention and effective treatment strategies. This review primarily focuses on therapeutic resistance in cutaneous melanoma. Evidence from other melanoma subtypes and, where relevant, other tumor types is included when it provides mechanistic or translational insight applicable to melanoma, and these findings are identified according to their disease context. Despite the rise in melanoma rates, several novel therapeutic strategies have mitigated the lethal effects of the disease. The most relevant of these are targeted therapies and immunotherapy, which have demonstrated improved overall survival compared with classical chemotherapy regimens. Melanoma, once considered one of the most feared malignancies, rapidly became a treatable disease in the era of precision oncology. More than a decade ago, the hallmark targeted therapy in melanoma was vemurafenib, which showed improved overall survival compared with dacarbazine, thus establishing [6]. Several agents targeting the B-Raf proto-oncogene, serine/threonine kinase (BRAF)/mitogen-activated protein kinase (MAPK) signaling pathway were subsequently developed, including dabrafenib and trametinib [7]. Importantly, combination regimens targeting multiple nodes within this pathway produced more durable responses and superior clinical efficacy compared with monotherapy approaches.

Following the advent of targeted therapies in melanoma, a new therapeutic avenue emerged with immunotherapy, which proved particularly useful for patients with melanomas lacking mutations amenable to targeted treatment. Notably, the first reported success of immune checkpoint inhibitors (ICIs) came in patients with metastatic melanoma, in whom the use of ipilimumab demonstrated a significant increase in overall survival [8]. In parallel with advances in targeted therapy, several other agents were subsequently shown to confer clinical benefit in patients with melanoma, including nivolumab, pembrolizumab, and combination regimens such as nivolumab plus ipilimumab, as well as newer immune checkpoint inhibitors targeting alternative pathways, such as relatlimab [9,10,11,12,13]. Together, these therapies fundamentally reshaped the therapeutic landscape of advanced melanoma.

Despite these remarkable therapeutic advances, resistance to both targeted therapy and immunotherapy remains the principal obstacle to durable disease control in melanoma. Most patients treated with MAPK pathway inhibitors ultimately experience disease relapse through adaptive and acquired mechanisms that restore oncogenic signaling or activate compensatory pathways [14]. Phenotypic plasticity further fuels this process, whereby melanoma cells transition between proliferative microphthalmia-associated transcription factor (MITF)-high and AXL receptor tyrosine kinase (AXL)-high states, a shift that has been shown to predict early therapeutic failure [15]. Resistance to ICIs likewise arises through tumor-intrinsic alterations, including mutations in Janus kinase 1/2 (JAK1/2) and beta-2-microglobulin (B2M) that impair interferon signaling and antigen presentation, as well as broader immune-evasive transcriptional programs identified in patient-derived tumors [16,17]. In addition to tumor-intrinsic mechanisms, tumor-extrinsic factors also contribute to therapeutic failure, with the gut microbiome emerging as a critical modulator of response to programmed cell death protein 1 (PD-1) blockade and specific commensal bacterial profiles correlating with improved antitumor immunity [18,19].

Accordingly, this review aims to integrate current insights into the molecular and immunologic mechanisms underlying melanoma resistance with emerging therapeutic strategies designed to overcome them. Particular emphasis is placed on novel immune checkpoint combinations, downstream pathway inhibition, and cell-based immunotherapies. By linking advances in tumor biology with innovations in rational drug design, biomarker discovery, and therapeutic engineering, this review highlights how mechanistic insights can inform the development of next-generation therapies capable of achieving more durable disease control. Importantly, resistance mechanisms are considered alongside their measurable biomarkers, potential therapeutic interventions, available clinical evidence, and current limitations. This approach also helps distinguish established clinical strategies from those that remain investigational or have shown limited clinical benefit.

### Methodology

The literature search was conducted using PubMed, Scopus, and ClinicalTrials.gov, with the final search completed in February 2026. Search terms included combinations of “melanoma,” “therapeutic resistance,” “drug resistance,” “BRAF,” “MEK,” “MAPK,” “PI3K/AKT/mTOR,” “immunotherapy resistance,” “immune checkpoint inhibitors,” “metabolic resistance,” and “emerging therapies.” Primary studies and melanoma-specific evidence were prioritized whenever available. Reviews were used mainly for background information and to identify additional relevant studies. Evidence from other tumor types was included selectively when it helped explain a biological mechanism or therapeutic approach relevant to melanoma. ClinicalTrials.gov was also used to verify ongoing and completed trials discussed in the review.

## 2. Molecular Basis of Melanoma and Drivers of Therapeutic Resistance

### 2.1. Genetic Landscape and Signaling Pathways

Modern advances in molecular biology have substantially improved our understanding of melanoma pathogenesis, revealing a complex landscape of genetic alterations that converge on key signaling pathways regulating cell proliferation, survival, and therapeutic response. Among the most prevalent driver mutations are activating alterations in BRAF, NRAS proto-oncogene, GTPase (NRAS), and loss-of-function mutations in neurofibromin 1 (NF1), which occur in approximately 50%, 25%, and 15% of cutaneous melanomas, respectively [20]. The oncogenic effects of these mutations are primarily mediated through dysregulation of the MAPK signaling cascade, the central pathway governing melanoma proliferation and survival. The pathway consists of rat sarcoma (RAS) proteins, rapidly accelerated fibrosarcoma (RAF) kinases, mitogen-activated protein kinase kinase (MEK), and extracellular signal-regulated kinase (ERK), each transmitting proliferative signals that are generated at and relayed from the cell surface to the nucleus [20]. Under physiologic conditions, this pathway is initiated by the binding of a mitogen, hormone, or neurotransmitter to a receptor tyrosine kinase (RTK). The binding triggers ligand-induced dimerization of the RTKs, thereby activating RAS through a change from guanosine diphosphate (GDP) to guanosine triphosphate (GTP)-bound RAS at the plasma membrane via growth factor receptor-bound protein 2 (GRB2)/son of sevenless (SOS) [21]. Activated RAS orchestrates the assembly of a multi-protein signaling hub composed of RAF kinases, MEK1/2, ERK1/2, and associated scaffold proteins, thereby launching the MAPK signaling cascade. In parallel, activated RAS also enhances signaling through the phosphoinositide 3-kinase (PI3K)–AKT serine/threonine kinase (AKT) pathway, reinforcing pro-survival and proliferative signals [22].

Among the components of the MAPK pathway, BRAF represents the most frequently altered kinase in cutaneous melanoma. Patients with BRAF mutations tend to be younger compared with patients with other melanoma subtypes [23]. The BRAF V600E mutation leads to a disruption in autoinhibitory control at the adenosine triphosphate (ATP)-binding site and renders MEK phosphorylation and activation independent of RAS signaling. This, in turn, triggers sustained ERK-driven tumorigenesis through upregulation of cyclin D1 and activation of cyclin-dependent kinase 4 (CDK4) [24]. Another prevalent oncogenic mutation involves RAS proteins, which have been reported to be implicated in 30% of all human cancers. A substitution from glutamine at residue 61 to leucine in NRAS is the most frequently encountered RAS mutation in melanoma [25]. Mutations in codon 61, as described above, lead to an NRAS that is incapable of properly coordinating water molecules involved in the hydrolysis of GTP to GDP, effectively locking NRAS in the GTP-bound state [26]. In addition to activating mutations in BRAF and NRAS, loss of NF1 represents another major mechanism underlying constitutive MAPK pathway activation. NF1 encodes neurofibromin, a large multi-domain RAS GTPase-activating protein (GAP) with a central GAP-related domain (GRD) that is responsible for replacing GTP bound to RAS with GDP, thereby negatively regulating and attenuating the MAPK pathway [27]. Loss of NF1 constitutively leaves the MAPK pathway active. Notably, multiple studies have indicated that NF1 has RAS-independent functions that factor into its tumor-suppressive role, yet these remain poorly elucidated [28].

Beyond aberrant MAPK signaling, disruption of cell cycle regulatory pathways represents another critical event in melanoma carcinogenesis. There are multiple cell cycle regulators that are involved in DNA replication, including cyclins, cyclin-dependent kinases (CDKs), and CDK inhibitors [29]. Key cell cycle regulators include cyclin-dependent kinase inhibitor 2A (CDKN2A), which encodes the tumor suppressors p16INK4A and p14ARF, CDKs involved in the control of specific checkpoints of the cell cycle, and the tumor suppressor retinoblastoma protein (Rb) [30]. Dysfunction in the p16INK4A—cyclin D–cyclin-dependent kinase 4/6 (CDK4/6)—Rb pathway is common in both cutaneous and acral melanomas, with a prevalence of 68.8–82.7% [31]. Notably, cyclin D1 (CCND1), a G1 phase cell cycle regulator, is a known oncogenic factor whose amplification is involved in melanoma. Current data have shown that CCND1 amplification is likely mutually exclusive with BRAF, RAS, and NF1 mutations, which constitute the most common mutant genes in melanoma and hence may be deemed an independent genomic subtype in melanoma [32].

However, sustained tumor progression depends on additional genetic aberrations discussed above, particularly the disruption of key tumor suppressor pathways such as inactivation of cyclin D–CDK4/6 and loss of phosphatase and tensin homolog (PTEN) [33]. PTEN’s role in melanoma revolves around both its lipid phosphatase activity and its phosphatase-independent activity, such as its scaffolding functions [34] PTEN’s lipid phosphatase activity antagonizes PI3K through the dephosphorylation of phosphatidylinositol trisphosphate (PIP3) to phosphatidylinositol bisphosphate (PIP2) [23]. By limiting the membrane availability of PIP3, PTEN prevents the membrane localization and activation of pleckstrin homology (PH) domain-containing effectors such as 3-phosphoinositide-dependent protein kinase 1 (PDK1) and AKT and thereby suppresses downstream pathway signaling, limiting growth and proliferation [35].

### 2.2. Phenotypic Plasticity

Melanoma heterogeneity results from differential expression of molecules such as RTKs in melanoma subsets. For instance, AXL, a member of the TYRO3 protein tyrosine kinase (TYRO3), AXL receptor tyrosine kinase (AXL), and MER proto-oncogene tyrosine kinase (MERTK) receptor family, has been shown to play a role in promoting tumor cell invasion and metastasis when overexpressed [36]. The expression of Axl often seems to be inversely correlated with the expression levels of MITF [37]. Axl serves as a marker of poorly differentiated melanoma cells with low MITF expression levels that exhibit enhanced migratory and invasive behavior, whereas TYRO3 expression, another TAM member shown to be a positive regulator of MITF in melanoma, is more tightly linked with more lineage-committed tumors with a higher proliferative capacity in melanomas expressing this receptor [38]. Given its pivotal role in regulating melanoma cell differentiation and phenotype, MITF has been extensively studied as a key determinant of melanoma biology. MITF, a member of the basic helix-loop-helix leucine zipper family of transcription factors, is considered to be the master regulator of melanogenesis, as it upregulates the expression of multiple enzymes required for the various steps of this process. MITF can have tumor-promoting functions by directly regulating genes involved in cell cycle progression and DNA replication, such as cyclin B1 (CCNB1), cyclin-dependent kinase 2 (CDK2), and telomerase reverse transcriptase (TERT) [39]. Beyond its role in regulating cell cycle progression, MITF also influences melanoma cell migration and invasion. For instance, increased MITF expression has been shown to reduce diaphanous-related formin 1 (DIA1) expression, a protein responsible for cytoskeletal rearrangement through the control of actin polymerization, thereby reducing the invasiveness of melanoma cells [40]. These differential effects of MITF have led to the proposition of a model by Carreira et al. suggesting that different MITF expression levels promote either invasion, proliferation, or differentiation. Within this framework, diminished MITF expression characterizes a dedifferentiated, stem cell-like state marked by cell arrest in G1 and enhanced invasive capacity, while cells expressing intermediate levels of MITF are associated with proliferating cells. Elevated expression of MITF defines cells that are more differentiated, but that are G1 arrested [37]. Taken together, the inverse correlation between Axl expression and MITF expression, along with diminished MITF expression in cells characterized by a greater invasive capacity, further delineates two populations: an MITF-high proliferative phenotype and an AXL-high invasive phenotype.

The dynamic alterations in MITF expression that underlie these phenotypic states are closely associated with melanoma phenotypic switching, a process that shares functional and molecular characteristics with epithelial-to-mesenchymal transition (EMT). EMT enables polarized epithelial cells to undergo major biochemical changes and adopt a mesenchymal cell phenotype, and this process is regulated by multiple autonomous and paracrine signals [41]. This phenomenon has also been reported in non-epithelial cancers such as melanoma, where it is referred to as “phenotype switching” [41]. As previously mentioned, melanoma exists in two phenotypes: a poorly invasive but rapidly proliferating phenotype, currently referred to as “melanocytic”, and a highly invasive but slowly proliferative phenotype, now referred to as “undifferentiated”. Interestingly, evidence suggests that cells can alternate between the proliferative and invasive states, thereby driving drug resistance and tolerance. Studies have shown that proliferative melanoma cells can adopt invasive cell characteristics upon MAPK inhibition, indicating that phenotype switching might actually be promoted by targeted therapy itself [42]. This binary division is currently the subject of refinement through the identification of multiple additional cell states [43]. Research by Tsoi et al. elucidated four distinct melanoma states: “undifferentiated, neural crest (NC)-like, transitory, and melanocytic” [44].

### 2.3. Tumor Microenvironment

The tumor microenvironment (TME) plays a central role in melanoma biology and is increasingly recognized as a dynamic and heterogeneous system that shapes tumor initiation, progression, and immune interactions. Rather than acting as a passive scaffold, the melanoma TME actively participates in tumor evolution through interconnected cellular, molecular, and metabolic processes [45]. It is commonly characterized by hypoxia, acidosis, and a dense composition that includes stromal and immune cell populations, vascular and lymphatic networks, and a remodeled extracellular matrix (ECM) [45]. This ECM is enriched with cytokines, chemokines, and multiple growth factors that collectively shape tumor behavior and therapeutic responsiveness [46,47]. Notably, both the cellular composition and functional state of the TME vary not only across melanoma subtypes but also across stages of tumor development, reflecting continuous bidirectional crosstalk between malignant cells and their surrounding niche [48].

#### 2.3.1. Immunosuppressive Cell Populations in the Melanoma TME

A hallmark of the melanoma TME is the accumulation of immunosuppressive immune cell populations that facilitate immune evasion. These coexist alongside effector immune cells, including regulatory T cells (Tregs), tumor-associated macrophages (TAMs), and myeloid-derived suppressor cells (MDSCs) [47].

Tregs, characterized by expression of forkhead box P3 (FOXP3) and CD25, accumulate progressively in melanoma lesions and are most commonly found in peripheral blood, sentinel lymph nodes, and in tumor-infiltrating lymphocytes populations [49,50]. These cells exert immunosuppressive effects through multiple mechanisms, including secretion of interleukin-10 (IL-10) and transforming growth factor-β (TGF-β) and the generation of adenosine via CD39/CD73 enzymatic activity, which inhibits dendritic cell activation and effector T-cell function [49]. Distinct Treg subpopulations have been described in melanoma, with induced Tregs (CD39^+^CD73^+^) representing the most immunosuppressive fraction within tumor infiltrates [49]. Furthermore, macrophages are the most abundant immune cells within the melanoma TME. TAMs originate from both tissue-resident macrophages and bone marrow-derived monocytes and display marked phenotypic plasticity [51,52]. Although TAMs exist along numerous activation states, melanoma lesions are enriched in macrophages exhibiting immunosuppressive, M2-like features and characterized by the expression of arginase 1 (Arg1), CD206, and the secretion of IL-10, TGF-β, and vascular endothelial growth factor (VEGF) [53,54]. TAMs interact closely with melanoma cells and stromal components, thereby contributing to immune modulation, angiogenesis, and tissue remodeling [55]. Importantly, melanoma-derived metabolites such as lactate further define the TAM phenotype, thus promoting recruitment and polarization toward immunoregulatory states [54]. While less extensively studied in the literature, MDSCs are also functionally linked to the suppressive immune network operating within the melanoma TME. MDSCs are immature myeloid cells that expand in response to tumor-derived cytokines and metabolic stressors. In fact, they contribute to the suppression of T-cell responses through arginine depletion, reactive oxygen species production, and secretion of inhibitory cytokines, working in parallel with TAMs and Tregs [56].

#### 2.3.2. Cancer-Associated Fibroblasts (CAFs) and Extracellular Matrix (ECM) Remodeling

Beyond immune cell populations, non-immune stromal cells also play essential roles in shaping the melanoma TME, with CAFs playing a major role in regulating ECM remodeling and tumor progression. In healthy skin, reticular and papillary fibroblasts serve distinct roles in tissue homeostasis [46]. However, during melanoma development, fibroblasts adjacent to tumor cells, mostly reticular, undergo activation and transition into CAFs through epigenetic reprogramming and transcriptional changes rather than major genetic mutations [57,58]. Following activation, CAFs become major drivers of ECM remodeling through the secretion of collagens, fibronectin, proteoglycans, and matrix-modifying enzymes such as matrix metalloproteinases (MMPs) and a disintegrin and metalloproteinase (ADAM) family proteases. This leads to altered ECM composition, increased stiffness, and enhanced mechanical tension within the melanoma TME [58,59]. Pizzurro et al.’s 3D model demonstrated that fibroblast-rich regions exhibit more aligned and thickened collagen fibers, thus highlighting the essential role of CAFs in organizing melanoma tissue architecture [55]. In addition to remodeling the ECM, CAFs function as major signaling centers within the melanoma TME. In melanoma, CAFs secrete a wide array of cytokines and growth factors such as interleukin-1β (IL-1β), interleukin-6 (IL-6), interleukin-8 (IL-8), C-X-C motif chemokine ligand 12 (CXCL12), vascular endothelial growth factor-A (VEGF-A), hepatocyte growth factor (HGF), and fibroblast growth factor-2 (FGF-2) that influence melanoma cell behavior and immune cell recruitment [58,60]. Recent transcriptional profiling has identified distinct CAF subtypes in skin cancers, including matrix CAFs (mCAFs), immunomodulatory CAFs (iCAFs), and myofibroblast-like CAFs (myoCAFs), each having unique ECM and immune-related functions [61]. Among these subpopulations, iCAFs are enriched in melanoma and aggressive skin tumors, and exhibit increased expression of inflammatory cytokines and immunoregulatory molecules such as indoleamine 2,3-dioxygenase 1 (IDO1), hence highlighting their role in immune modulation within the TME [61].

#### 2.3.3. Metabolic Competition and Biochemical Features of the Melanoma TME

Metabolic competition is a key feature of the melanoma TME and profoundly affects how both tumor and immune cells function. Rapid tumor growth and aberrant vascularization lead to a hypoxic and acidic milieu, resulting in the accumulation of metabolic byproducts such as lactate [46]. Melanoma cells and CAFs both contribute to elevated lactate levels, which serve not only as metabolic waste but also as signaling molecules within the TME [62]. As a result, this promotes recruitment and polarization of macrophages toward immunoregulatory phenotypes and impairs effector T-cell proliferation and cytokine production, thus explaining how lactate accumulation alters immune cell behavior [54]. In addition, metabolic competition for essential nutrients further shapes immune activity. A prominent example is tryptophan catabolism, mediated by IDO1. IDO1 is usually expressed by tumor cells, CAFs, dendritic cells, and macrophages, and represents a key metabolic feature of the melanoma TME [61,63]. Tryptophan depletion together with accumulation of kynurenine metabolites suppresses T-cell proliferation while promoting regulatory immune phenotypes, thereby reinforcing immune tolerance within the TME [64]. Collectively, these metabolic challenges overlap with cellular and structural components of the TME, creating a highly dynamic and heterogeneous microenvironment. Through coordinated actions of immunosuppressive cell populations, CAF-driven ECM remodeling, and metabolic competition, the melanoma TME emerges as a central determinant of tumor–immune interactions and disease behavior, underscoring its importance as a focal point for biological and clinical application and therapeutics.

### 2.4. Implications for Drug Discovery

#### 2.4.1. Target Tractability (Kinases, Epigenetic Regulators, and Immune Checkpoints)

Recent advances in the understanding of melanoma biology have identified several molecular pathways for targeted therapeutic intervention. Among protein kinases, BRAF and its downstream effectors MEK1/2 and ERK1/2 represent the most clinically validated oncogenic targets. ATP-competitive BRAF inhibitors, including vemurafenib, dabrafenib, and encorafenib, have been clinically approved [20,21,24]. Although direct RAS inhibition remains pharmacologically challenging, downstream nodes, including MEK and ERK, remain tractable in these genetic contexts [27,28]. Dysregulation of the CDK4/6–Rb axis through CDKN2A loss, CDK4 amplification, or CCND1 gain further extends the kinase target space, with CDK4/6 inhibitors demonstrating preclinical and clinical activity in CDKN2A-altered melanoma [30,31]. Additionally, PTEN loss derepresses the PI3K/AKT/mechanistic target of rapamycin (mTOR) pathway, identifying AKT and mTOR as co-targets alongside MAPK inhibition [33,34]. The AXL receptor tyrosine kinase, upregulated in the drug-tolerant MITF-low melanoma phenotype, has emerged as a therapeutically relevant target at the intersection of tumor plasticity and immune evasion, with preclinical evidence supporting its combined inhibition with BRAF-targeted therapy to eliminate heterogeneous, drug-resistant cell populations [15,36,37,38]. Beyond kinase signaling, epigenetic regulators have emerged as an important class of therapeutic targets. The chromatin remodeling events that drive phenotypic plasticity in melanoma are mediated by histone deacetylases (HDACs) and bromodomain-containing proteins (BRDs), both of which are pharmacologically targetable [42,44,65]. HDAC inhibitors have been shown to modulate MAPK and immune signaling in preclinical melanoma models and exhibit the potential to reverse drug-tolerant epigenetic states, supporting their evaluation in combination with targeted or immune therapies [66]. Enhancer of zeste homolog 2 (EZH2), a histone methyltransferase that controls gene silencing programs associated with the invasive melanoma phenotype, represents an additional epigenetically tractable target for which clinical-grade pharmacological tools are available [42,65].

In addition to targeting intrinsic tumor signaling pathways, therapeutic strategies have increasingly focused on restoring antitumor immunity through immune checkpoint inhibitors. Immune checkpoint molecules constitute the most clinically advanced target class in melanoma. PD-1/programmed death-ligand 1 (PD-L1) and cytotoxic T-lymphocyte-associated protein 4 (CTLA-4) have been validated through regulatory approvals of pembrolizumab, nivolumab, ipilimumab, and the lymphocyte activation gene-3 (LAG-3) inhibitor relatlimab [8,9,10,11,12,13]. The immunosuppressive circuitry described above reveals further checkpoint-like targets operative in tumors that have escaped canonical blockade, including the IDO1-tryptophan catabolism axis, the CD39/CD73/adenosine pathway, and TGF-β signaling, each of which impairs effector T-cell function within the melanoma TME [67,68]. Collectively, the convergence of kinase oncoproteins, epigenetic regulators, and immune checkpoint molecules defines a multi-dimensional target landscape that reflects the biological complexity of melanoma and provides the mechanistic foundation for multi-target therapeutic strategies.

#### 2.4.2. Biomarker-Driven Stratification for Therapy

The heterogeneous molecular landscape of melanoma necessitates biomarker-guided patient stratification to rationalize therapeutic selection and anticipate resistance trajectories. BRAF mutational status remains the foundational genomic stratification biomarker, directing patients with V600 mutations toward MAPK inhibitor-based therapy or immunotherapy-first approaches based on tumor context and disease characteristics [20,23]. However, within BRAF-mutant melanoma, co-occurring alterations substantially modulate response durability and resistance patterns: PTEN loss, CDKN2A inactivation, and secondary NRAS mutations are associated with more aggressive resistance trajectories, arguing for comprehensive genomic profiling at baseline to stratify patients by resistance risk [30,33,69,70]. Beyond genomic mutation status, phenotypic and microenvironmental features offer additional stratification dimensions. The MITF/AXL expression ratio constitutes a functionally informative biomarker: tumors exhibiting low MITF and high AXL expression at baseline harbor an intrinsically drug-tolerant phenotype with a propensity for early acquired resistance to both MAPK-targeted therapies and immune checkpoint inhibitors, whereas MITF-high tumors may be more responsive to differentiation-directed interventions [15,36,37,38]. At the level of the tumor microenvironment, quantification of immunosuppressive cell infiltrates, including Tregs, MDSCs, and M2-polarized TAMs, alongside assessment of IDO1 expression and CD39/CD73 pathway activation may help identify immune-excluded or immune-desert tumor contextures in which conventional PD-1 blockade is unlikely to achieve durable disease control [61,63,64].

In addition to these emerging molecular and microenvironmental biomarkers, PD-L1 expression, tumor mutational burden (TMB), and tumor-infiltrating lymphocyte (TIL) density are widely investigated biomarkers that provide complementary but incomplete prognostic or predictive information [71]. PD-L1 expression is limited by spatial and temporal heterogeneity and is not routinely used as a standalone marker for treatment selection in melanoma [71]. TMB captures neoantigen burden but requires standardized assessment methodologies and is insufficient in isolation to distinguish responsive from resistant tumors [72]. TIL density reflects immune contexture but must be interpreted alongside T-cell exhaustion markers, including PD-1, T-cell immunoglobulin and mucin-domain-containing-3 (TIM-3), and LAG-3, to assess functional immune engagement and distinguish between inflamed and dysfunctional immune phenotypes [72]. Collectively, these data support the advancement toward integrated biomarker panels that combine tumor-intrinsic genomic features, phenotypic state markers, and TME immune contexture profiling, enabling a more refined and dynamic approach to patient stratification beyond single-marker assessments [73,74].

#### 2.4.3. Rationale for Combination Drug Design

The integrated molecular portrait presented previously provides strong mechanistic underpinnings for combination therapeutic strategies in melanoma, built upon three converging principles. First, oncogenic pathway redundancy and inter-pathway crosstalk preclude durable control by single-agent approaches. The MAPK and PI3K/AKT/mTOR pathways are coupled through shared upstream activators, most notably mutant RAS, which simultaneously engages both signaling axes [75,76]. Single-agent MAPK inhibition frequently selects for compensatory PI3K/AKT activation, and PTEN loss further derepresses this bypass axis, enabling sustained tumor proliferation despite upstream BRAF or MEK blockade [75,76,77]. This molecular architecture necessitates co-targeting of multiple pathway nodes to achieve adequate and durable suppression of oncogenic output, as supported by preclinical evidence and limited early clinical investigation of combined MAPK and PI3K/AKT/mTOR inhibition [78,79]. Second, the bidirectional interplay between tumor cell-intrinsic signaling and the immunosuppressive tumor microenvironment creates a co-dependency that neither modality can overcome alone. MAPK pathway activation contributes to immune evasion by upregulating PD-L1 expression and impairing antigen presentation, while IDO1 upregulation, Treg accumulation, and lactate-driven macrophage polarization within the TME collectively insulate tumor cells from immune-mediated elimination [61,63,64,74]. Combinations that simultaneously disable oncogenic signaling and restore immune surveillance are therefore mechanistically coherent, addressing complementary and interdependent resistance mechanisms. Third, phenotypic plasticity and epigenetic escape constitute a fundamental resistance mechanism that neither kinase inhibition nor immunotherapy addresses in isolation. MAPK inhibition itself enriches for the MITF-low, AXL-high drug-tolerant population, which exhibits increased invasiveness, metabolic reprogramming toward oxidative phosphorylation, and reduced immunogenicity, thereby seeding subsequent resistance to both targeted and immune therapies [65].

## 3. Mechanisms of Resistance to Current Therapies

Despite major advances in targeted therapy and immunotherapy, melanoma frequently develops resistance through genetic, epigenetic, metabolic, and microenvironmental adaptation. This section outlines the principal mechanisms by which melanoma cells evade current therapies and ultimately drive disease relapse.

### 3.1. Resistance to BRAF/MEK/ERK-Targeted Therapy

**Figure 1:**
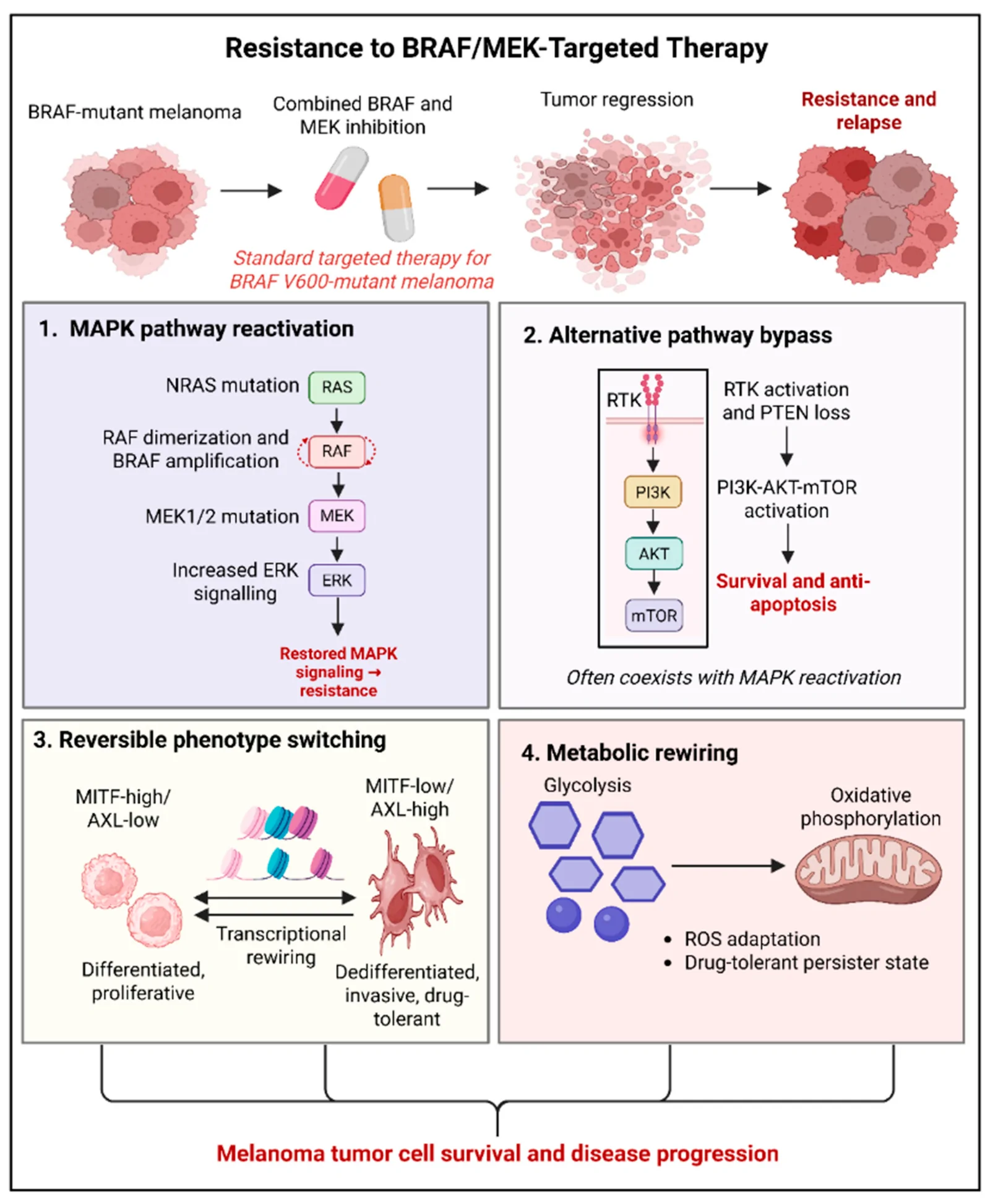
Mechanisms of resistance to BRAF/MEK-targeted therapy in melanoma. This figure summarizes the major mechanisms underlying resistance to combined BRAF and MEK inhibition, the current standard targeted therapy for BRAF V600-mutant melanoma. After initial tumor regression, resistance and relapse may occur through four major mechanisms: MAPK pathway reactivation, including NRAS mutation, RAF dimerization and BRAF amplification, and MEK1/2 mutation, which restore downstream ERK signaling; alternative pathway bypass, including RTK activation and PTEN loss with compensatory PI3K-AKT-mTOR signaling that promotes survival and anti-apoptosis; reversible phenotype switching, characterized by transition from a MITF-high/AXL-low differentiated proliferative state to a MITF-low/AXL-high dedifferentiated, invasive, drug-tolerant state; and metabolic rewiring, in which melanoma cells shift toward oxidative phosphorylation and adaptive survival programs, including reactive oxygen species management and drug-tolerant persister states. Together, these pathways support melanoma tumor cell survival and disease progression. Abbreviations: AXL, AXL receptor tyrosine kinase; BRAF, B-Raf proto-oncogene serine/threonine kinase; ERK, extracellular signal-regulated kinase; MAPK, mitogen-activated protein kinase; MEK, mitogen-activated protein kinase kinase; MITF, microphthalmia-associated transcription factor; mTOR, mechanistic target of rapamycin; NRAS, neuroblastoma RAS viral oncogene homolog; PI3K, phosphoinositide 3-kinase; PTEN, phosphatase and tensin homolog; RAF, rapidly accelerated fibrosarcoma kinase; ROS, reactive oxygen species; RTK, receptor tyrosine kinase. Created in BioRender. El darzi, R. (2026) https://BioRender.com/tvnvncs, accessed on 12 August 2026.

Treating patients with BRAF-mutant melanoma with BRAF inhibitors alone or in combination with MEK inhibitors usually leads to rapid tumor regression. However, these responses are often not durable, with many patients eventually experiencing relapse or disease progression. Clinical and molecular analyses indicate that resistance arises through multiple, often overlapping mechanisms, including MAPK pathway reactivation, bypass signaling, phenotypic plasticity, metabolic rewiring, and immune escape (Figure 1). Resistance may be primary, whereby tumors fail to respond from the outset, or acquired, whereby resistance develops after a period of sensitivity. In both scenarios, resistance to treatment demonstrates the remarkable capacity of melanoma cells to adapt and persist, leading to relapse [69,70].

#### 3.1.1. MAPK Pathway Reactivation

Among acquired resistance mechanisms, MAPK pathway reactivation is one of the most frequently observed routes of therapeutic escape following BRAF and MEK inhibition. However, as discussed, these responses remain limited, and many patients suffer from relapse and disease progression. Biopsies from post-treatment patients demonstrate that resistance to therapy is not driven by a single molecular alteration, but is a complex process that reflects the multilayered adaptive response of tumor cells to therapy. These mechanisms include, but are not limited to, genetic and signaling alterations, transcriptional reprogramming, and metabolic adaptations [69,70]. The most frequently observed mechanism of resistance involves the reactivation of MAPK signaling, even in the presence of the combined BRAF and MEK inhibition. Secondary alterations that restore ERK activity have been identified in clinical meta-analyses and longitudinally sampled tumors [69]. For instance, acquired NRAS mutations have been identified; these promote RAF dimerization and further downstream signaling [70]. MEK1/2 mutations that confer constitutive kinase activity or reduced sensitivity to MEK inhibitors have also been described [70]. These alterations resulted in restoring proliferative and survival signaling. Furthermore, BRAF gene amplification also increases the tumor’s oncogenic signaling output, allowing it to overcome the pharmacologic inhibition through dosage effects. This is due to the tumor’s capacity to generate multiple copies of the mutant BRAF gene. Hence, even if a drug inhibits a portion of the BRAF protein, the increased abundance of mutant BRAF overwhelms the inhibitor, resulting in the dosage effect [69]. Moreover, another prominent resistance mechanism involves the emergence of BRAF splice variants that lack the RAS-binding domain. These splice variants no longer require RAS to regulate activation and dimerization; thus, they are constitutively active and preferentially form dimers. This alteration renders first-generation RAF inhibitors, such as vemurafenib and dabrafenib, ineffective, as these were designed to inhibit monomeric BRAF. Such variants have been identified in patients with tumors progressing on therapy and are thought to account for a substantial fraction of acquired resistance cases [80]. Even upon the administration of dual BRAF/MEK blockade, restored ERK phosphorylation has been reported, highlighting the central role of MAPK reactivation in therapeutic escape [70,75].

#### 3.1.2. Alternative Pathway Bypass

In addition to MAPK pathway reactivation, melanoma cells can further acquire resistance by activating and engaging in alternative survival pathways, thus reducing their dependence on ERK signaling. For instance, some resistant tumors have been shown to activate receptor tyrosine kinase signaling, thereby activating the PI3K/AKT pathway and its downstream effector mTOR [75,76]. Further loss of PTEN, a major negative regulator of the signaling axis discussed above, enhances cell survival and proliferation. Activation of this signaling axis promotes anti-apoptotic signals and supports proliferation even during suppression of the MAPK pathway. It is important to note that within the same resistant tumor cells, MAPK reactivation and bypass signaling frequently coexist [75,76]. This highlights the redundant and adaptable nature of signaling networks rather than a switch from one pathway to another.

#### 3.1.3. Epigenetic and Transcriptional Mechanisms

Beyond genetic and signaling alterations, transcriptional reprogramming and epigenetic plasticity are major drivers of therapeutic resistance in melanoma. MITF is a master regulator of melanocyte identity and melanoma cell state. MITF expression exists along a continuum, with its level determining melanoma cell phenotype and behavior. Hence, melanoma cells exist along a balance between a MITF-high, AXL-low state, which is a proliferative state, and a MITF-low, AXL-high state, which refers to an invasive, drug-resistant state [65]. Targeted therapy selectively enriches for the latter phenotype by eliminating sensitive, MITF-high cells while allowing intrinsically resistant MITF-low cells to dominate the tumor population [65]. This selection for the MITF-low, AXL-high state induces dedifferentiation, reduced proliferation, and increased invasiveness, thus making such cells less vulnerable to treatment. This phenotype switching is not mediated by mutations but rather by chromatin remodeling, altered transcription factor networks, and reversible epigenetic modifications, thereby enabling rapid adaptation to therapeutic stress [65]. Such states contribute significantly to intratumoral heterogeneity and relapse, as observed in post-treatment samples.

#### 3.1.4. Metabolic Rewiring

Blockade of the MAPK pathway places melanoma cells under substantial metabolic stress, necessitating adaptive changes in energy production and redox homeostasis. Under basal conditions, sensitive melanoma cells rely on glycolysis as a fast energy source; however, resistant cells switch to mitochondrial oxidative phosphorylation (OXPHOS) [65]. OXPHOS serves as a more efficient method for ATP production under stress, as it is less dependent on MAPK-driven glycolytic pathways. This is also accompanied by an increased capacity to manage reactive oxygen species, which are generated during OXPHOS [65]. These changes are coordinated with the transcriptional state of the cell, thus leading to the survival of drug-tolerant populations. Hence, transcriptional analyses indicate that melanoma cells that survive initial MAPK inhibition adopt distinct adaptive states, including altered metabolic programs, stress response signaling, and reversible dedifferentiation, that favor their selection [65].

### 3.2. Resistance to Immune Checkpoint Inhibitors

**Figure 2:**
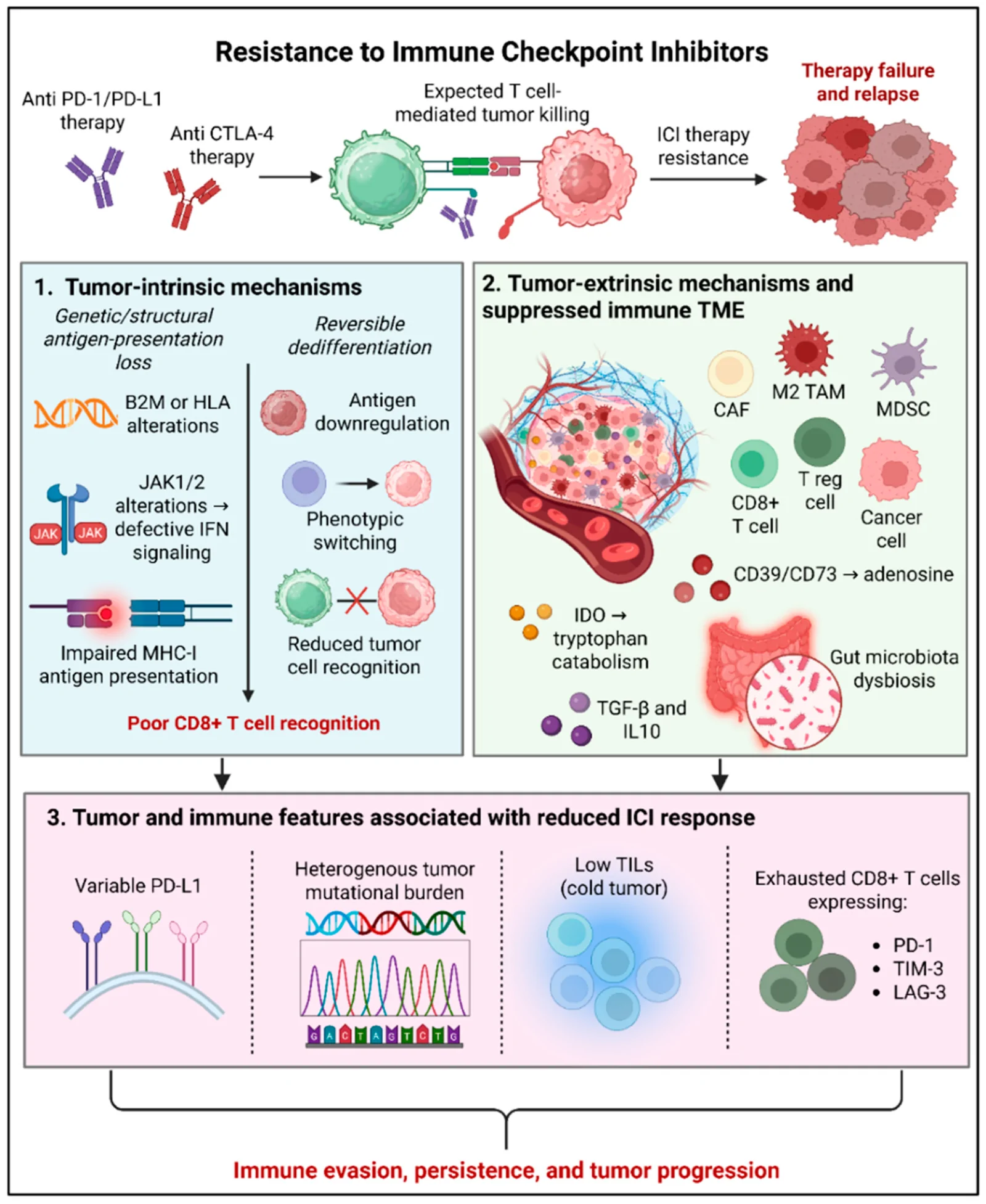
Mechanisms of resistance to immune checkpoint inhibitors in melanoma. This figure illustrates major mechanisms of resistance to immune checkpoint inhibitor (ICI) therapy in melanoma. Following anti-PD-1/PD-L1 or anti-CTLA-4 therapy, resistance may arise through tumor-intrinsic mechanisms, tumor-extrinsic immunosuppressive mechanisms within the tumor microenvironment (TME), and associated tumor and immune features linked to reduced ICI response. Tumor-intrinsic mechanisms are divided into genetic/structural antigen presentation loss—including B2M or HLA alterations, JAK1/2 alterations with defective interferon signaling, and impaired MHC-I antigen presentation—and reversible dedifferentiation, including antigen downregulation and phenotypic switching, both of which reduce CD8^+^ T-cell recognition. Tumor-extrinsic mechanisms include immunosuppressive stromal and immune cell populations, metabolic suppression, and gut microbiota dysbiosis. Collectively, these processes promote immune evasion, persistence, and tumor progression. **Abbreviations:** B2M, beta-2-microglobulin; CAF, cancer-associated fibroblast; CD8, cluster of differentiation 8; CTLA-4, cytotoxic T-lymphocyte-associated protein 4; HLA, human leukocyte antigen; ICI, immune checkpoint inhibitor; IDO, indoleamine 2,3-dioxygenase; IFN, interferon; IL, interleukin; JAK, Janus kinase; LAG-3, lymphocyte activation gene-3; MDSC, myeloid-derived suppressor cell; MHC-I, major histocompatibility complex class I; PD-1, programmed cell death protein 1; PD-L1, programmed death-ligand 1; TAM, tumor-associated macrophage; TGF-β, transforming growth factor beta; TIL, tumor-infiltrating lymphocyte; TIM-3, T-cell immunoglobulin and mucin-domain containing-3; TME, tumor microenvironment; Treg, regulatory T cell. Created in BioRender. El darzi, R. (2026) https://BioRender.com/esx30r7, accessed on 12 August 2026.

Resistance to ICIs in melanoma treatment arises from two major categories of mechanisms: tumor-intrinsic and tumor-extrinsic mechanisms, both of which contribute to immune evasion (Figure 2). Tumor-intrinsic factors involve the tumor’s ability to alter antigen presentation and interferon signaling pathways, whereas tumor-extrinsic mechanisms are associated with the immunosuppressive tumor microenvironment. Several biomarkers of resistance exist, including PD-L1 expression variability, TMB heterogeneity, and TIL infiltration [71,73].

#### 3.2.1. Tumor Intrinsic and Extrinsic Mechanisms

Tumor-intrinsic mechanisms mainly involve defects in antigen presentation machinery. B2M plays a key role in major histocompatibility complex (MHC) class I folding and surface antigen presentation to CD8^+^ T cells. Alterations involving B2M, including loss-of-function mutations and genomic loss, have been associated with resistance to immune checkpoint blockade. More broadly, genetic changes affecting antigen presentation and interferon-γ signaling pathways have been identified in approximately one quarter of ICI-resistant melanoma cases [73]. Furthermore, mutations in JAK1 or JAK2 disrupt intracellular signaling and downstream cascades that promote antigen presentation and immune activation. Lastly, melanoma dedifferentiation and phenotypic plasticity further contribute to immune resistance by downregulating wild-type melanocytic differentiation antigens and MHC-I expression, limiting effective immune recognition [73]. Tumor-extrinsic mechanisms involve the immunosuppressive TME, which inhibits antitumor activity through molecular and cellular signaling [71,74]. Cytokines such as TGF-β and IL-10 play a crucial role in immunosuppression by inhibiting effector T cells and promoting the expansion of regulatory T cells. Immunosuppressive cells, including M2-polarized tumor-associated macrophages (M2-TAMs) and myeloid-derived suppressor cells (MDSCs), are key components of the immunosuppressive TME, exerting their effects through the secretion of inhibitory cytokines and the expression of immune checkpoint ligands [74]. Metabolic and microbial dysregulation have also been shown to increase resistance to immune checkpoint inhibitors. For example, upregulation of the CD39/CD73/adenosine pathway and IDO-dependent tryptophan catabolism within the TME impairs T-cell activation and effector function [71]. Interestingly, even distant from the tumor site, specific gut microbiota alterations have been implicated in melanoma resistance to ICIs [81]. Sivan et al. demonstrated that transferring gut microbiota from mice with slow-growing melanoma to mice with aggressive tumors enhanced T-cell infiltration and improved the response to anti-PD-1 therapy [82].

#### 3.2.2. Biomarkers of Resistance

Several biomarkers have been investigated as predictors of resistance to ICIs and as tools to optimize treatment in melanoma. Among the most studied is PD-L1 expression in tumor cells; however, its clinical utility remains limited, as it does not reliably predict treatment response in all melanoma patients [72,83]. Another important factor is TMB, with higher mutation loads generating more neoantigens for presentation to T cells. However, TMB assessment requires standardization across laboratories, and its use in the clinical setting remains promising but not fully established [71]. Furthermore, “cold” melanomas, characterized by low lymphocyte infiltration, have been associated with decreased response to immune checkpoint inhibitors. Low TIL infiltration can result from low TMB and reduced neoantigen presentation, leading to diminished recognition by the immune system [83]. Nevertheless, this is not always predictive of response. In tumors with abundant TILs, CD8^+^ T cells repeatedly stimulated by tumor neoantigens may become dysfunctional, entering a state called “exhaustion,” characterized by elevated expression of inhibitory receptors such as PD-1, TIM-3, and LAG-3, reducing the effectiveness of immunotherapy [71,83]. These resistance mechanisms have prompted the development of emerging therapeutic strategies designed to overcome MAPK pathway reactivation, bypass signaling, immune exhaustion, metabolic adaptation, and tumor microenvironment-mediated resistance.

## 4. Emerging Pharmaceutical Approaches to Overcome Resistance

**Figure 3:**
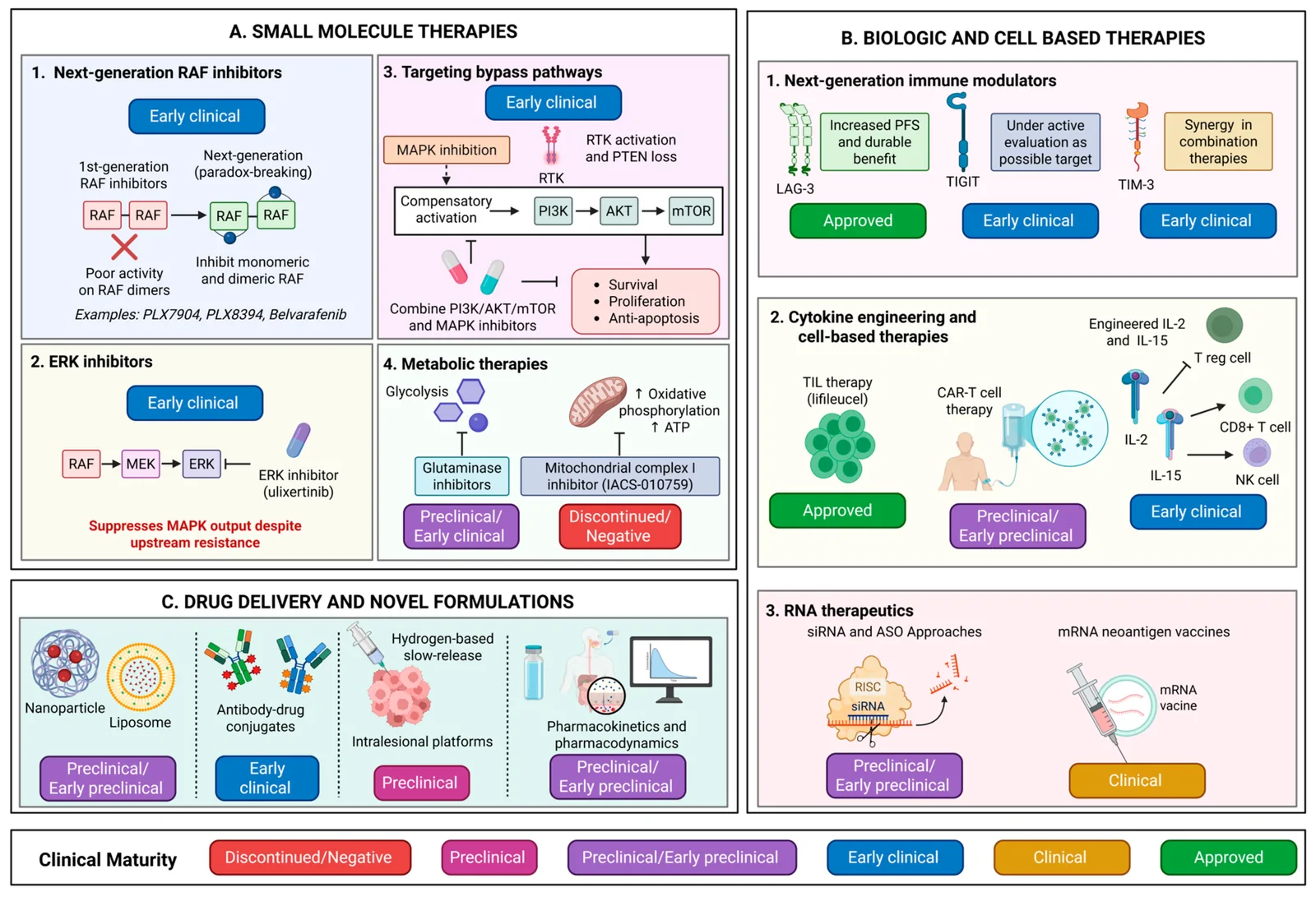
Emerging therapeutic strategies and drug delivery approaches in melanoma. This figure summarizes selected therapeutic strategies proposed to overcome resistance in melanoma, grouped into small-molecule therapies, biologic and cell-based therapies, and drug delivery/novel formulation platforms. Small-molecule approaches include next-generation RAF inhibitors, ERK inhibition, combined MAPK and PI3K/AKT/mTOR pathway targeting, and metabolic therapies. Biologic and cell-based approaches include next-generation immune modulators, cytokine engineering and cell-based therapies, and RNA therapeutics. Drug delivery approaches include nanoparticle and liposomal systems, antibody-drug conjugates, hydrogel-based slow-release or intralesional platforms, and pharmacokinetic/pharmacodynamic optimization strategies. Clinical maturity labels indicate the current highest level of evidence in melanoma and range from discontinued/negative, preclinical, preclinical/early clinical, early clinical, and clinical, to approved. These categories are intended to distinguish investigational from clinically established approaches and do not imply equivalent efficacy across strategies. Abbreviations: AKT, protein kinase B; ASO, antisense oligonucleotide; CAR-T, chimeric antigen receptor T-cell; ERK, extracellular signal-regulated kinase; IL, interleukin; LAG-3, lymphocyte activation gene-3; MAPK, mitogen-activated protein kinase; mTOR, mechanistic target of rapamycin; PD-1, programmed cell death protein 1; PFS, progression-free survival; PI3K, phosphoinositide 3-kinase; PTEN, phosphatase and tensin homolog; RAF, rapidly accelerated fibrosarcoma kinase; RTK, receptor tyrosine kinase; siRNA, small interfering RNA; TIL, tumor-infiltrating lymphocyte; TIM-3, T-cell immunoglobulin and mucin-domain containing-3; TIGIT, T-cell immunoreceptor with immunoglobulin and ITIM domain. Created in BioRender. El darzi, R. (2026) biorender.com/unbfjgl, accessed on 12 August 2026.

The diverse mechanisms underlying resistance to targeted therapy and immunotherapy have prompted the development of emerging therapeutic strategies designed to restore pathway suppression, overcome bypass signaling, enhance antitumor immune activity, and limit adaptive tumor plasticity. These approaches include next-generation small-molecule inhibitors, biologic and cell-based therapies, RNA-based therapeutics, and novel drug delivery platforms (Figure 3). The current level of evidence, developmental status, and principal limitations of selected strategies are also summarized in Table 1.

### 4.1. Small-Molecule Therapies

#### 4.1.1. Next-Generation RAF Inhibitors

Resistance to first-generation RAF inhibitors frequently results from restoration of MAPK signaling through RAF dimerization and pathway reactivation mechanisms [84,85]. To overcome this limitation, next-generation RAF inhibitors have been designed to suppress signaling from both monomeric and dimeric RAF complexes while minimizing paradoxical MAPK activation in wild-type cells [86]. PLX8394, a next-generation BRAF inhibitor that avoids paradoxical RAF/MEK/ERK pathway activation, was evaluated in a phase I/II study of patients with refractory BRAF-mutant solid tumors. In the phase II cohort, 18 patients were enrolled, including five patients with BRAF V600-mutant melanoma; among 10 evaluable patients across tumor types, three had stable disease, although melanoma-specific outcomes were not reported separately [87]. Lifirafenib (BGB-283), an investigational RAF family kinase inhibitor, was evaluated in a first-in-human phase I dose escalation and dose expansion study of patients with BRAF- or RAS-mutated advanced solid tumors. Clinical responses were observed in patients with BRAF-mutant melanoma [88]. Belvarafenib (HM95573/GDC-5573), an oral type II pan-RAF kinase inhibitor, was evaluated in a phase I study of patients with advanced solid tumors harboring BRAF, KRAS, or NRAS mutations, with antitumor activity reported in patients with RAS- or RAF-mutant tumors [89]. Despite these advances, incomplete suppression of all RAF signaling nodes and adaptive pathway rewiring continue to limit long-term efficacy, supporting combination strategies with downstream MAPK inhibitors [85,90].

#### 4.1.2. ERK Inhibitors

Reactivation of ERK signaling represents a convergent resistance mechanism following RAF or MEK inhibition, making ERK a rational downstream therapeutic target [91]. Direct inhibition of ERK1/2 suppresses MAPK pathway output irrespective of upstream resistance alterations, including RAF dimerization and MEK reactivation [91,92]. Ulixertinib, a first-in-class ERK inhibitor, has demonstrated antitumor activity [92] in patients with MAPK-driven cancers who had progressed on prior RAF or MEK inhibitor therapies [93]. However, adaptive resistance to ERK inhibition can develop through secondary ERK mutations or engagement of alternative signaling circuits, indicating that ERK inhibitors may be most effective when used in rational combination regimens [91,93].

#### 4.1.3. Targeting Bypass Pathways

Although direct ERK inhibition addresses MAPK pathway reactivation, compensatory activation of parallel survival pathways remains an important mechanism limiting durable therapeutic responses. Compensatory activation of parallel survival pathways, particularly the PI3K/AKT/mTOR axis, represents a major mechanism of escape from MAPK-directed therapies [77]. Crosstalk between MAPK and PI3K signaling enables tumor cells to maintain proliferative and anti-apoptotic signaling under selective pressure from RAF, MEK, or ERK inhibition [78]. Combined targeting of PI3K or AKT with MAPK inhibitors has shown enhanced pathway suppression and improved antitumor activity in preclinical studies and limited early clinical investigation [78,79]. In addition, phosphoinositide regulators such as SH2-containing inositol phosphatase 1 and 2 (SHIP1/2) modulate AKT pathway activity, and early translational studies suggest that targeting SHIP-dependent signaling may influence sensitivity to MAPK-targeted therapy [94].

#### 4.1.4. Metabolic Therapies

Consistent with the metabolic rewiring described above, therapy-resistant tumors often exhibit metabolic reprogramming characterized by increased reliance on OXPHOS and glutamine metabolism [95,96]. MAPK inhibitor-resistant tumor subpopulations frequently adopt an OXPHOS-high phenotype, creating a therapeutic vulnerability to mitochondrial complex I inhibitors such as IACS-010759 [97]. Preclinical studies demonstrate that OXPHOS inhibition preferentially impairs drug-resistant tumor cells and can enhance the antitumor activity of MAPK-targeted therapies when used in combination [95,96]. However, this preclinical promise has not translated into clinical benefit with IACS-010759. In two phase I trials involving patients with refractory acute myeloid leukemia or advanced solid tumors, IACS-010759 showed a narrow therapeutic index with dose-limiting elevations in blood lactate and neurotoxicity. No recommended phase II dose was established, antitumor activity was limited at tolerated doses, and both trials were discontinued [98]. In parallel, BRAF inhibitor-resistant melanoma cells can develop increased dependence on glutamine metabolism, and inhibition of glutaminolysis has demonstrated activity against these resistant populations in preclinical models [99]. Combined targeting of metabolic dependencies and oncogenic signaling pathways may therefore constrain adaptive resistance by limiting both signaling and energetic escape mechanisms [97,99].

### 4.2. Biologic and Cell-Based Therapies

Advances in the understanding of melanoma immunobiology have led to the development of biologic and cell-based therapies that have substantially improved clinical outcomes. These novel therapeutic strategies enhance antitumor immune responses through distinct mechanisms and have significantly improved survival outcomes in patients with advanced melanoma [100]. Historically, advanced melanoma was associated with a poor prognosis, with a median overall survival of approximately six to nine months; however, the introduction of modern immunotherapy fundamentally changed the therapeutic landscape of advanced melanoma [101].

#### 4.2.1. Next-Generation Immune Modulators

Among the biologic therapies, ICIs have become the cornerstone of treatment for advanced melanoma. Ten-year results from CheckMate 067 confirmed the durability of this advantage, with a median overall survival of 71.9 months with nivolumab plus ipilimumab, compared with 36.9 months with nivolumab alone and 19.9 months with ipilimumab alone [102]. Despite these advances, up to 65% of patients show primary resistance to PD-1 inhibitors, and approximately 43% of responders develop acquired resistance within three years [103]. Next-generation immune checkpoints, including LAG-3, TIM-3, and T-cell immunoreceptor with Ig and ITIM domains (TIGIT), are being targeted to overcome these limitations [104,105]. LAG-3 suppresses T-cell function via major histocompatibility complex class II (MHC-II) binding. In RELATIVITY-047, the approved fixed-dose combination of nivolumab plus relatlimab significantly improved progression-free survival (PFS) compared with nivolumab alone. With longer follow-up, median PFS was 10.2 versus 4.6 months, while the subsequent OS analysis showed a clinically meaningful but not statistically significant improvement in survival [106]. TIGIT, frequently co-expressed with LAG-3 on tumor-infiltrating lymphocytes (TILs), drives T-cell exhaustion; anti-TIGIT combinations with PD-1/PD-L1 blockade are under active early-phase evaluation [107]. TIM-3, expressed broadly on innate and adaptive immune cells, contributes to T-cell exhaustion and PD-1 resistance; combination trials, including cobolimab plus dostarlimab, suggest limited monotherapy activity but potential synergy in combination regimens [105].

#### 4.2.2. Cytokine Engineering and Cell-Based Therapies

Beyond immune checkpoint modulation, additional immunotherapeutic strategies seek to enhance antitumor immunity by directly enhancing immune cell activation and function.

(a) Engineered interleukin-2 (IL-2) and interleukin-15 (IL-15)

Cytokine-based immunotherapy was pioneered by high-dose IL-2 (aldesleukin). It was the first agent to achieve durable complete responses in advanced melanoma but was limited by severe systemic toxicity. Second-generation engineered IL-2 variants, including bempegaldesleukin, were developed with the aim of improving selectivity and reducing toxicity. However, the phase III PIVOT IO 001 trial did not meet its primary endpoints of objective response rate, progression-free survival, or overall survival with bempegaldesleukin plus nivolumab compared with nivolumab alone. Grade 3–4 treatment-related adverse events were also more frequent with the combination (21.7% vs. 11.5%) [108] IL-15 superagonists such as ALT-803 (N-803) selectively expand CD8^+^ memory T cells and natural killer (NK) cells without promoting Tregs, and are under investigation in combination with anti-PD-1 and adoptive cell therapies [109,110,111,112].

(b) TIL Therapy: Lifileucel

Approved by the U.S. Food and Drug Administration (FDA) in February 2024, lifileucel is the first TIL therapy for unresectable or metastatic melanoma refractory to prior ICI or BRAF/MEK inhibitor therapy. The five-year analysis of the C-144-01 trial demonstrated a 31.4% objective response rate (ORR), a median duration of response of 36.5 months, and 31.3% of responders remaining in response at five years [113,114]. In ICI-naive patients (IOV-COM-202 trial), lifileucel plus pembrolizumab achieved a 65.2% ORR with a 30.4% complete response rate [114]. The ongoing phase III TILVANCE-301 trial is evaluating lifileucel combined with pembrolizumab versus pembrolizumab alone as first-line therapy [114]. Key challenges include tumor tissue availability, TIL heterogeneity, an immunosuppressive TME, and the high cost and personalization burden of therapy [115].

(c) Chimeric antigen receptor T-cell (CAR-T) Therapy Adaptations

While CAR-T therapy achieves approximately 80–90% durable responses in certain hematologic malignancies, solid tumors, including melanoma, present significant barriers: poor trafficking, dense extracellular matrix, metabolic suppression, and antigen escape [116,117]. Ongoing engineering strategies include locoregional delivery, chemokine receptor modification through C-C motif chemokine receptor 2b (CCR2b), C-X-C motif chemokine receptor 2 (CXCR2), or C-X-C motif chemokine receptor 6 (CXCR6), multi-target platforms such as bispecific CAR-T and bispecific T-cell engager (BiTE)-secreting CAR-T cells, and armored constructs incorporating IL-15 or dominant-negative TGF-β receptors to enhance persistence and overcome the immunosuppressive TME [116,117].

#### 4.2.3. RNA Therapeutics

In addition to cell-based immunotherapies, RNA-based therapeutics have emerged as a versatile area for modulating oncogenic signaling, immune responses, and mechanisms of therapeutic resistance.

(a) Small interfering RNA (siRNA) and antisense oligonucleotide (ASO) Approaches

RNA-based therapeutics enable selective silencing of oncogenic drivers and resistance mediators. Key targets include signal transducer and activator of transcription 3 (STAT3) (danvatirsen ASO), TGF-β2, Kirsten rat sarcoma viral oncogene homolog (KRAS), and ribonucleotide reductase regulatory subunit M2 (RRM2), which was evaluated as one of the systemic siRNA targets in humans through a melanoma clinical trial [118,119] STP355, a siRNA-nanoparticle silencing TGF-β1 and vascular endothelial growth factor receptor 2 (VEGFR2), reduced tumor burden and enhanced T-cell and NK-cell infiltration in B16 melanoma models, with additive effects when combined with PD-L1 or CTLA-4 blockade [120]. ASOs targeting metastasis-associated lung adenocarcinoma transcript 1 (MALAT1) inhibit BRAF expression and can bypass MEK inhibitor resistance in MAPK-driven melanoma [121].

(b) Messenger RNA (mRNA) Neoantigen Vaccines

Melanoma’s high mutational burden makes it well suited for personalized mRNA neoantigen vaccine strategies. BioNTech’s IVAC MUTANOME trial (NCT02035956) demonstrated potent de novo neoepitope-specific T-cell responses with sustained recurrence-free survival [122,123]. Moderna’s mRNA-4157 (V940) combined with pembrolizumab in the randomized phase 2b KEYNOTE-942 trial showed a 44% reduction in the risk of relapse or death (hazard ratio (HR) = 0.56, *p* = 0.0266) versus pembrolizumab alone in resected stage III/IV melanoma [124]. Challenges include mRNA instability, limited cellular uptake, and the immunosuppressive TME; lipid nanoparticle (LNP) delivery platforms and combination with ICIs are key strategies to address these [125].

### 4.3. Drug Delivery and Novel Formulations

To maximize therapeutic efficacy while minimizing systemic toxicity, considerable efforts have focused on the development of novel drug delivery platforms discussed below.

#### 4.3.1. Nanoparticles and Liposomes

Nanoparticle-based systems—including liposomes, LNPs, and polymeric nanoparticles—improve bioavailability, enable targeted tumor delivery, and reduce toxicity for both siRNA and tyrosine kinase inhibitor (TKI) payloads [126]. Liposomal TKIs (cabozantinib, sorafenib, erlotinib) achieve enhanced tumor localization and reduced systemic toxicity in preclinical models [127]. The first-in-class phase I trial by Ribas et al. demonstrated tolerable systemic siRNA delivery via nanoparticles in cancer patients, while liposomal protease-activated receptor-1 (PAR-1) siRNA markedly suppressed melanoma growth and metastasis in murine models [128].

#### 4.3.2. Antibody-Drug Conjugates (ADCs)

ADCs combine tumor-targeting monoclonal antibodies with potent cytotoxic payloads via specialized linkers, enabling selective cancer cell killing with reduced systemic toxicity [66,129,130,131,132]. At the time of this review, no ADC has received FDA approval specifically for the treatment of melanoma. Several ADCs are being investigated in this setting, targeting glycoprotein nonmetastatic melanoma protein B (gpNMB) (glembatumumab vedotin), human epidermal growth factor receptor 3 (HER3) (patritumab deruxtecan; NCT06172478), AXL (BA3011; NCT03425279), receptor tyrosine kinase-like orphan receptor 2 (ROR2) (ozuriftamab vedotin; NCT03504488), and human epidermal growth factor receptor 2 (HER2) (RC48, NCT06003231) [132,133,134]. Key challenges include tumor antigen heterogeneity, off-target toxicities, linker instability, and drug resistance via efflux pumps or antigen downregulation [135,136,137,138].

#### 4.3.3. Intralesional Platforms and Pharmacokinetic/Pharmacodynamic (PK/PD) Optimization

Intralesional delivery overcomes the systemic toxicity and immune clearance that limit intravenous administration of cytokines and oncolytic viruses. Hydrogel-based slow-release matrices protect cytokines, including IL-2 and interleukin-12 (IL-12), and oncolytic viruses, including talimogene laherparepvec (T-VEC), from degradation, enable controlled intratumoral release, and sustain immune activation while dampening early antiviral responses [139,140,141]. Thermosensitive hydrogels (e.g., F127-g-Gelatin) that sustain co-releases of BRAF inhibitors and anti-PD-1 antibodies reversed acquired resistance in BRAF V600-mutant preclinical models [142]. Long-acting formulations (NT-I7 interleukin-7 (IL-7) fusion protein, polymeric drug conjugates, microneedle patches) and stimuli-responsive nanocarriers further improve PK/PD profiles by maintaining therapeutic drug levels above resistance thresholds and improving intratumoral penetration [143,144,145,146,147,148].

## 5. Imaging and Biomarker Tools to Monitor Resistance

As emerging therapies continue to expand, there is a growing need for tools to capture treatment response and resistance beyond conventional radiographic assessment. Molecular imaging and liquid biopsy approaches offer complementary strategies to monitor pathway activity, immune engagement, and tumor evolution throughout therapy. However, most of the pathway- and immune-specific radiotracers discussed below remain in the preclinical or investigational stage and have not been incorporated into routine clinical practice.

### 5.1. Positron Emission Tomography/Single-Photon Emission Computed Tomography (PET/SPECT) Radiotracers

Molecular imaging with PET and SPECT provides a noninvasive approach to assess pathway activity, target expression, and immune contexture during melanoma therapy. Beyond standard ^18F-fluorodeoxyglucose (^18F-FDG) imaging, investigational radiotracers are being developed to evaluate oncogenic signaling and immune checkpoint expression [148,149,150].

Reactivation of the MAPK pathway is a central mechanism of resistance to BRAF and MEK inhibitors in melanoma. Secondary NRAS mutations, BRAF amplification or splice variants, MEK mutations, and upstream receptor tyrosine kinase signaling can restore ERK activity despite upstream blockade. [151,152,153]. Radiolabeled kinase inhibitors have therefore been explored as pharmacodynamic probes of MAPK pathway activity and drug–target engagement. A radiolabeled trametinib analog has demonstrated target-related tumor uptake in preclinical models [154], while other kinase inhibitor-based PET tracers provide proof-of-concept evidence that oncogenic pathway engagement can be assessed in vivo [155].

Immuno-PET provides a complementary approach to evaluating spatial heterogeneity in immune checkpoint expression. Radiolabeled PD-L1-targeting agents, including ^89Zr-atezolizumab, have demonstrated heterogeneous tumor uptake and associations with PD-L1 expression in early human studies across solid tumors, including melanoma [156]. Smaller engineered tracers such as 18F-BMS-986192 allow more rapid imaging and have also demonstrated quantifiable, heterogeneous PD-L1 uptake in early clinical studies [157]. Beyond PD-L1, CD8-targeted PET agents are being investigated to assess cytotoxic T-cell infiltration, while granzyme B tracers aim to measure effector immune activity [158,159,160].

Overall, MAPK- and immune-targeted PET tracers remain largely investigational and currently serve primarily as exploratory or pharmacodynamic research tools. Although they may provide information on spatial heterogeneity and target engagement, limited clinical validation and availability currently preclude their routine use for treatment selection or adaptation.

### 5.2. Liquid Biopsy and Genomic Biomarkers

Liquid biopsy provides a minimally invasive approach for longitudinal assessment of melanoma evolution and treatment response. Circulating tumor DNA (ctDNA) can reflect tumor burden and detect emerging resistance-associated alterations, including secondary NRAS and MAPK-pathway changes during targeted therapy [161,162]. Early ctDNA clearance has been associated with more favorable outcomes, whereas persistent or increasing ctDNA is generally associated with poorer prognosis [163,164,165]. Extracellular vesicles provide another investigational source of circulating biomarkers. In melanoma, exosomal miRNAs and proteins have been linked to resistance mechanisms; for example, exosomal miR-19a has been associated with vemurafenib resistance, while circulating exosomal PD-L1 has been associated with impaired antitumor immunity and poorer response to PD-1 blockade [165,166].

Collectively, these approaches have important prognostic and monitoring potential; however, they are not yet sufficiently validated to guide routine treatment switching in the absence of clinical or radiographic progression. By integrating genomic indicators such as ctDNA with exosomal miRNA and protein signatures, liquid biopsy may complement conventional tissue biopsy and clinical assessment by providing additional information on tumor evolution and emerging resistance [167].

### 5.3. Translational Integration

Translational integration seeks to combine established clinical markers with emerging molecular and circulating biomarkers to characterize treatment response and evolving resistance over time.

#### 5.3.1. Using Biomarkers for Real-Time Therapy Adjustment

Different classes of systemic therapy have distinct kinetics and mechanisms of response, making longitudinal biomarker assessment potentially useful during treatment [168]. In routine practice, readily available markers such as serum lactate dehydrogenase (LDH) provide prognostic information and correlate with outcomes in advanced melanoma. This is reflected by the inclusion of LDH in staging for stage IV disease and its utility in monitoring response and progression over time [169]. In parallel, inflammatory markers linked to tumor burden and immune activity, including IL-6 and C-reactive protein (CRP), have been associated with response and survival following checkpoint blockade. Because individual biomarkers are unlikely to capture the full heterogeneity of melanoma, multimarker approaches may provide complementary information, although most emerging circulating biomarker panels are not yet validated as independent triggers for treatment escalation or switching [169].

In addition to routine laboratory parameters, a key translational advance is the use of blood-based “liquid biopsy” biomarkers that can be sampled repeatedly to reflect the evolving tumor landscape. Circulating tumor cells (CTCs) provide both quantitative and qualitative information, including their presence and abundance, as well as surface marker profiles that may shift under therapeutic pressure [170,171]. PD-L1 expression on CTCs has also been explored as a noninvasive marker of dynamic tumor–immune interactions, although CTC-based measurements remain investigational and are not currently recommended for routine treatment selection [172].

Exploratory studies have also suggested metabolic differences in melanoma CTCs, including relative enrichment of oxidative phosphorylation, although the clinical significance of these findings remains uncertain [173].

#### 5.3.2. Early Resistance Detection and Adaptive Therapeutic Strategies

Resistance in melanoma is not a single event but often a gradual transition driven by tumor plasticity, immune escape, and TME stress. Phenotypic switching and stress-associated translation reprogramming, including reduced MITF expression and ATF4-driven adaptive programs, have been linked to drug-resistant melanoma states [174].

Early detection of resistance can be strengthened by combining tumor-intrinsic biomarkers with immune context biomarkers. Although its measurement is technically variable and remains largely exploratory, TMB has been associated with response to checkpoint inhibition in melanoma, supporting the broader concept that integrating genomic features with immune signatures may better identify tumors that are more likely to respond [175]. Other well-described resistance-associated alterations include WNT/β-catenin activation, PTEN loss, JAK1/2 alterations, and defects in antigen presentation such as B2M loss.

Overall, integrating routinely available clinical markers, such as LDH and CRP, with immune response kinetics and liquid biopsy measures, such as CTC burden and PD-L1 expression, may provide additional information on evolving tumor biology when interpreted alongside established clinical and radiographic assessment. Most of these emerging biomarkers are not yet validated as independent triggers for treatment escalation or switching. This approach may also help reduce the clinical impact of resistance mechanisms driven by phenotypic plasticity and microenvironmental stress programs [176,177].

## 6. Future Directions and Clinical Translation

These advances in therapeutic development and resistance monitoring may support future biomarker-guided treatment strategies in melanoma, although many of these approaches still require prospective clinical validation. Following resistance to ICIs, several studies have explored the potential of combining immunotherapy, particularly ICIs, with targeted therapies such as BRAF and MEK inhibitors to overcome melanoma resistance. However, significant challenges remain. In a multicenter, double-blind, placebo-controlled trial evaluating the efficacy of this combination, patients with BRAFV600-mutant advanced melanoma were randomized to receive either placebo plus vemurafenib (BRAF inhibitor) and cobimetinib (MEK inhibitor) or atezolizumab (anti-PD-L1) combined with vemurafenib and cobimetinib. Initial results showed improved progression-free survival (PFS) in the combination group compared with targeted therapy alone, but no significant difference in overall survival (OS) was observed, indicating that further follow-up is required (median OS: 39.0 months with atezolizumab vs. 25.8 months in the control group; HR 0.84, *p* = 0.14) [178].

Another important consideration in BRAF V600-mutant metastatic melanoma is the sequence of immunotherapy and BRAF/MEK-targeted therapy. DREAMseq showed improved two-year overall survival when nivolumab plus ipilimumab was given first, followed by dabrafenib plus trametinib at progression, supporting an immunotherapy-first approach [179]. SECOMBIT provided complementary prospective evidence and evaluated a short course of targeted therapy before immunotherapy [180]. Targeted therapy may still be considered when rapid disease control is needed, particularly in patients with highly symptomatic or rapidly progressive disease [181]. These sequential approaches are distinct from concurrent triplet therapy, such as the IMspire150 regimen, in which immunotherapy and BRAF/MEK inhibition are administered together.

In order to minimize the severity and prevalence of adverse events in melanoma-resistant patients, precision medicine approaches using multi-omic profiling that integrate genomic, transcriptomic, and proteomic data are needed. Several predictive models have been developed. A Neoantigen Presentation Score, which considers neoantigen burden, immune-related resistance mechanisms, and human leukocyte antigen (HLA) allele-specific loss of heterozygosity (LOH), showed a stronger statistical association with ICI response (*p* = 0.002) than TMB (*p* = 0.049) and other single-gene or expression biomarkers [182]. Deep learning approaches are also being investigated for the prediction of immunotherapy response in melanoma. One such model, AMU (attention mechanism model for melanoma immUnotherapy), uses mRNA expression profiles and showed promising performance in its reported dataset [183]. However, these models remain exploratory, and their clinical use will require external validation, assessment of calibration and clinical utility, and evaluation in larger and more diverse patient populations.

Despite these advances, translational challenges remain, such as the higher incidence of adverse events with multi-drug regimens and the complexity of designing adaptive clinical trials that can optimize treatment sequencing and dosing [184,185]. Similarly, artificial intelligence (AI)-based biomarker discovery shows promise but faces obstacles including data variability, regulatory constraints, and limited accessibility [185]. Addressing these barriers is essential to translate predictive insights into safe and effective treatments.

## 7. Conclusions

Melanoma has evolved from a disease with a dismal prognosis to one that can increasingly be treated with targeted and immune-based interventions, yet therapeutic resistance remains its defining clinical obstacle. As reviewed herein, resistance reflects a multilayered adaptive process spanning genetic, epigenetic, metabolic, and microenvironmental dimensions. MAPK pathway reactivation, PI3K/AKT bypass signaling, phenotypic plasticity, and TME-mediated immunosuppression collectively enable tumor cells to evade both targeted therapies and immune recognition. Tumor-intrinsic immune evasion through impaired antigen presentation further compounds the inadequacy of checkpoint blockade.

Nevertheless, next-generation RAF and ERK inhibitors, rational co-targeting of bypass pathways, novel checkpoint combinations, and metabolic vulnerability-based strategies are expanding the therapeutic resources. Advances in PET/SPECT imaging, ctDNA, and liquid biopsy are enabling dynamic resistance monitoring, while multi-omic profiling and AI-driven biomarker models promise to refine patient stratification. Overcoming melanoma resistance will ultimately require adaptive, biomarker-guided frameworks that account for the spatial and temporal heterogeneity of the disease.

## Figures and Tables

**Table 1:** Selected therapeutic strategies to overcome resistance in melanoma and their current level of evidence.

Strategy	Target/Mechanism	Disease Setting	Evidence Level	Trial Phase	Trial Identifier	Current Status	Principal Limitation
Nivolumab + relatlimab	Dual PD-1/LAG-3 checkpoint blockade	Previously untreated unresectable or metastatic melanoma	Positive randomized clinical evidence	Phase II/III	RELATIVITY-047; NCT03470922	Approved fixed-dose combination; improved PFS versus nivolumab alone	Resistance still occurs; OS evidence is less definitive than the PFS benefit
Lifileucel TIL therapy	Adoptive transfer of autologous tumor-infiltrating lymphocytes	Advanced melanoma after prior systemic therapy; first-line combination under study	Established clinical activity; ongoing randomized evaluation	Phase II; ongoing Phase III	C-144-01 (NCT02360579); IOV-COM-202 (NCT03645928); TILVANCE-301 (NCT05727904)	FDA approved for previously treated unresectable or metastatic melanoma; first-line combination remains investigational	Tumor tissue requirements, manufacturing complexity, TIL heterogeneity, immunosuppressive TME, and cost
Bempegaldesleukin + nivolumab	Engineered IL-2 pathway agonism plus PD-1 blockade	Previously untreated unresectable or metastatic melanoma	Negative randomized evidence	Phase III	PIVOT IO 001; NCT03635983	Phase III trial did not meet ORR, PFS, or OS endpoints	Higher grade 3-4 treatment-related toxicity without efficacy advantage
mRNA-4157 (V940) + pembrolizumab	Personalized mRNA neoantigen vaccination plus PD-1 blockade	Resected high-risk stage III/IV melanoma	Positive randomized clinical signal	Phase 2b	KEYNOTE-942; NCT03897881	Investigational; improved recurrence-free outcomes versus pembrolizumab alone	Manufacturing and delivery complexity, mRNA stability, and immunosuppressive TME
PLX8394	Next-generation BRAF inhibition designed to limit paradoxical MAPK activation	Refractory or relapsed BRAF-mutant solid tumors; melanoma subgroup included	Early clinical evidence	Phase I/II	PLX120-03; NCT02428712	Investigational	Limited melanoma-specific clinical data and uncertain durability
Lifirafenib (BGB-283)	RAF-family kinase inhibition	BRAF- or RAS-mutated advanced solid tumors, including melanoma	Early clinical evidence	Phase I	NCT02610361	Investigational	Early-phase evidence; melanoma-specific efficacy and durability remain uncertain
Belvarafenib (HM95573/GDC-5573)	Type II pan-RAF/RAF-dimer inhibition	BRAF- or RAS-mutated advanced solid tumors, including melanoma	Early clinical evidence	Phase I	NCT02405065; NCT03118817	Investigational	Early-phase evidence and potential for adaptive or acquired RAF-pathway resistance
Ulixertinib (BVD-523)	Direct ERK1/2 inhibition downstream of RAF/MEK	MAPK-mutant advanced solid tumors; separately evaluated in metastatic uveal melanoma	Early clinical evidence	Phase I; Phase II	NCT01781429; NCT03417739	Investigational	Adaptive signaling and secondary ERK alterations can limit activity; uveal melanoma phase II showed limited efficacy
MAPK + PI3K/AKT pathway inhibition	Concurrent blockade of MAPK signaling and compensatory PI3K/AKT survival signaling	BRAF-mutant or BRAF-wild-type advanced melanoma and other solid tumors	Early clinical evidence	Phase I/I-II	NCT01902173	Investigational	Toxicity, drug–drug interactions, and limited clinical activity have constrained development
IACS-010759	Mitochondrial complex I/oxidative phosphorylation inhibition	Advanced solid tumors and relapsed/refractory AML; translational relevance to OXPHOS-dependent resistant states	Negative early clinical evidence	Phase I	NCT03291938; NCT02882321	Both phase I programs discontinued; no recommended Phase II dose established	Narrow therapeutic index, elevated lactate, neurotoxicity, and limited antitumor activity
CAR-T adaptations	Engineered T-cell targeting with trafficking, multi-target, or armored constructs	Solid tumors including melanoma	Preclinical/early translational	—	—	Investigational	Poor trafficking, dense ECM, metabolic suppression, antigen heterogeneity, and antigen escape
STP355 siRNA nanoparticle	Simultaneous silencing of TGF-β1 and VEGFR2	B16 melanoma models	Preclinical	Preclinical	—	Experimental	Evidence remains confined to preclinical melanoma models
MALAT1 antisense oligonucleotides	MALAT1 suppression affecting BRAF/MAPK signaling	MAPK-driven melanoma	Preclinical/mechanistic	Preclinical	—	Experimental	Clinical efficacy, delivery, and safety remain unestablished
Antibody-drug conjugates	Targeted cytotoxic delivery against gpNMB, HER3, AXL, ROR2, or HER2	Melanoma	Early clinical/investigational	Early clinical/ongoing	NCT06172478; NCT03425279; NCT03504488; NCT06003231	No FDA-approved ADC specifically for melanoma	Antigen heterogeneity, off-target toxicity, linker instability, antigen loss, and drug efflux
Hydrogel-based BRAF inhibitor + anti-PD-1 delivery	Sustained locoregional co-delivery of targeted therapy and immunotherapy	BRAF V600-mutant melanoma models	Preclinical	Preclinical	—	Experimental	Clinical translation and reproducibility in patients remain unestablished
Atezolizumab + vemurafenib + cobimetinib	Concurrent PD-L1 blockade with BRAF/MEK inhibition	Previously untreated BRAF V600-mutant advanced melanoma	Randomized Phase III evidence	Phase III	IMspire150; NCT02908672	Improved PFS; no statistically significant OS improvement in the reported follow-up	Treatment complexity and toxicity; OS benefit not established
Immunotherapy-first sequencing	Nivolumab/ipilimumab followed by dabrafenib/trametinib at progression	BRAF V600-mutant metastatic melanoma	Prospective randomized sequencing evidence	Phase III	DREAMseq/EA6134; NCT02224781	Supports an immunotherapy-first sequence for most patients	Targeted therapy may still be preferred when rapid disease control is required
Short targeted-therapy induction before immunotherapy	Brief BRAF/MEK inhibition followed by ipilimumab/nivolumab	Untreated BRAF V600-mutant metastatic melanoma	Prospective randomized clinical evidence	Phase II	SECOMBIT; NCT02631447	Investigational sequencing strategy	Optimal patient selection and sequencing remain unresolved

Abbreviations: ADC, antibody-drug conjugate; AML, acute myeloid leukemia; BRAF, B-Raf proto-oncogene; CAR-T, chimeric antigen receptor T cell; ECM, extracellular matrix; FDA, U.S. Food and Drug Administration; ICI, immune checkpoint inhibitor; IL, interleukin; LAG-3, lymphocyte activation gene-3; MAPK, mitogen-activated protein kinase; ORR, objective response rate; OS, overall survival; PD-1, programmed cell death protein 1; PD-L1, programmed death-ligand 1; PFS, progression-free survival; PI3K, phosphoinositide 3-kinase; TIL, tumor-infiltrating lymphocyte; TME, tumor microenvironment; VEGFR2, vascular endothelial growth factor receptor 2.

## Data Availability

No new data were created or analyzed in this study. Data sharing does not apply to this article.

## References

[1] Arnold M., Singh D., Laversanne M., Vignat J., Vaccarella S., Meheus F., Cust A.E., de Vries E., Whiteman D.C., Bray F. (2022). Global Burden of Cutaneous Melanoma in 2020 and Projections to 2040. JAMA Dermatol..

[2] Sung H., Ferlay J., Siegel R.L., Laversanne M., Soerjomataram I., Jemal A., Bray F. (2021). Global Cancer Statistics 2020: GLOBOCAN Estimates of Incidence and Mortality Worldwide for 36 Cancers in 185 Countries. CA Cancer J. Clin..

[3] Siegel R.L., Kratzer T.B., Giaquinto A.N., Sung H., Jemal A. (2025). Cancer statistics, 2025. CA Cancer J. Clin..

[4] Wang M., Gao X., Zhang L. (2025). Recent global patterns in skin cancer incidence, mortality, and prevalence. Chin. Med. J..

[5] De Pinto G., Mignozzi S., La Vecchia C., Levi F., Negri E., Santucci C. (2024). Global trends in cutaneous malignant melanoma incidence and mortality. Melanoma Res..

[6] Wolchok J.D., Chiarion-Sileni V., Gonzalez R., Rutkowski P., Grob J.J., Cowey C.L., Lao C.D., Wagstaff J., Schadendorf D., Ferrucci P.F. (2017). Overall Survival with Combined Nivolumab and Ipilimumab in Advanced Melanoma. N. Engl. J. Med..

[7] Robert C., Karaszewska B., Schachter J., Rutkowski P., Mackiewicz A., Stroiakovski D., Lichinitser M., Dummer R., Grange F., Mortier L. (2015). Improved overall survival in melanoma with combined dabrafenib and trametinib. N. Engl. J. Med..

[8] Hodi F.S., O’Day S.J., McDermott D.F., Weber R.W., Sosman J.A., Haanen J.B., Gonzalez R., Robert C., Schadendorf D., Hassel J.C. (2010). Improved survival with ipilimumab in patients with metastatic melanoma. N. Engl. J. Med..

[9] Wolchok J.D., Chiarion-Sileni V., Gonzalez R., Grob J.J., Rutkowski P., Lao C.D., Cowey C.L., Schadendorf D., Wagstaff J., Dummer R. (2022). Long-Term Outcomes with Nivolumab Plus Ipilimumab or Nivolumab Alone Versus Ipilimumab in Patients with Advanced Melanoma. J. Clin. Oncol..

[10] Tawbi H.A., Schadendorf D., Lipson E.J., Ascierto P.A., Matamala L., Castillo Gutiérrez E., Rutkowski P., Gogas H.J., Lao C.D., De Menezes J.J. (2022). Relatlimab and Nivolumab versus Nivolumab in Untreated Advanced Melanoma. N. Engl. J. Med..

[11] Robert C., Schachter J., Long G.V., Arance A., Grob J.J., Mortier L., Daud A., Carlino M.S., McNeil C., Lotem M. (2015). Pembrolizumab versus Ipilimumab in Advanced Melanoma. N. Engl. J. Med..

[12] Robert C., Long G.V., Brady B., Dutriaux C., Maio M., Mortier L., Hassel J.C., Rutkowski P., McNeil C., Kalinka-Warzocha E. (2015). Nivolumab in previously untreated melanoma without BRAF mutation. N. Engl. J. Med..

[13] Larkin J., Chiarion-Sileni V., Gonzalez R., Grob J.J., Cowey C.L., Lao C.D., Schadendorf D., Dummer R., Smylie M., Rutkowski P. (2015). Combined Nivolumab and Ipilimumab or Monotherapy in Untreated Melanoma. N. Engl. J. Med..

[14] Tripathi R., Liu Z., Jain A., Lyon A., Meeks C., Richards D., Liu J., He D., Wang C., Nespi M. (2020). Combating acquired resistance to MAPK inhibitors in melanoma by targeting Abl1/2-mediated reactivation of MEK/ERK/MYC signaling. Nat. Commun..

[15] Müller J., Krijgsman O., Tsoi J., Robert L., Hugo W., Song C., Kong X., Possik P.A., Cornelissen-Steijger P.D., Geukes Foppen M.H. (2014). Low MITF/AXL ratio predicts early resistance to multiple targeted drugs in melanoma. Nat. Commun..

[16] Zaretsky J.M., Garcia-Diaz A., Shin D.S., Escuin-Ordinas H., Hugo W., Hu-Lieskovan S., Torrejon D.Y., Abril-Rodriguez G., Sandoval S., Barthly L. (2016). Mutations Associated with Acquired Resistance to PD-1 Blockade in Melanoma. N. Engl. J. Med..

[17] Lim S.Y., Shklovskaya E., Lee J.H., Pedersen B., Stewart A., Ming Z., Irvine M., Shivalingam B., Saw R.P.M., Menzies A.M. (2023). The molecular and functional landscape of resistance to immune checkpoint blockade in melanoma. Nat. Commun..

[18] Matson V., Fessler J., Bao R., Chongsuwat T., Zha Y., Alegre M.L., Luke J.J., Gajewski T.F. (2018). The commensal microbiome is associated with anti-PD-1 efficacy in metastatic melanoma patients. Science.

[19] Gopalakrishnan V., Spencer C.N., Nezi L., Reuben A., Andrews M.C., Karpinets T.V., Prieto P.A., Vicente D., Hoffman K., Wei S.C. (2018). Gut microbiome modulates response to anti-PD-1 immunotherapy in melanoma patients. Science.

[20] Smalley K.S., Haass N.K., Brafford P.A., Lioni M., Flaherty K.T., Herlyn M. (2006). Multiple signaling pathways must be targeted to overcome drug resistance in cell lines derived from melanoma metastases. Mol. Cancer Ther..

[21] Fang X., Wang S., Fu S. (2025). Dissecting the MAPK signaling landscape in malignant melanoma: From BRAF and NRAS mutations to precision combination therapies. Front. Cell Dev. Biol..

[22] Fensterle J. (2006). A trip through the signaling pathways of melanoma. J. Dtsch. Dermatol. Ges..

[23] Kim H.J., Kim Y.H. (2024). Molecular Frontiers in Melanoma: Pathogenesis, Diagnosis, and Therapeutic Advances. Int. J. Mol. Sci..

[24] Hirai N., Hatanaka Y., Hatanaka K.C., Uno Y., Chiba S.I., Umekage Y., Minami Y., Okumura S., Ohsaki Y., Sasaki T. (2021). Cyclin-dependent kinase 4 upregulation mediates acquired resistance of dabrafenib plus trametinib in BRAF V600E-mutated lung cancer. Transl. Lung Cancer Res..

[25] Inamdar G.S., Madhunapantula S.V., Robertson G.P. (2010). Targeting the MAPK pathway in melanoma: Why some approaches succeed and other fail. Biochem. Pharmacol..

[26] Jenkins R.W., Sullivan R.J. (2016). NRAS mutant melanoma: An overview for the clinician for melanoma management. Melanoma Manag..

[27] Ballester R., Marchuk D., Boguski M., Saulino A., Letcher R., Wigler M., Collins F. (1990). The NF1 locus encodes a protein functionally related to mammalian GAP and yeast IRA proteins. Cell.

[28] Berry D., Moldoveanu D., Rajkumar S., Lajoie M., Lin T., Tchelougou D., Sakthivel S., Sharon I., Bernard A., Pelletier S. (2025). The NF1 tumor suppressor regulates PD-L1 and immune evasion in melanoma. Cell Rep..

[29] Leal-Esteban L.C., Fajas L. (2020). Cell cycle regulators in cancer cell metabolism. Biochim. Biophys. Acta Mol. Basis Dis..

[30] Yang T.T., Yu S., Ke C.K., Cheng S.T. (2023). The Genomic Landscape of Melanoma and Its Therapeutic Implications. Genes.

[31] Foffano L., Cucciniello L., Nicolò E., Migliaccio I., Noto C., Reduzzi C., Malorni L., Cristofanilli M., Gerratana L., Puglisi F. (2025). Cyclin-dependent kinase 4 and 6 inhibitors (CDK4/6i): Mechanisms of resistance and where to find them. Breast.

[32] Liu J., Lin J., Wang X., Zheng X., Gao X., Huang Y., Chen G., Xiong J., Lan B., Chen C. (2022). CCND1 Amplification Profiling Identifies a Subtype of Melanoma Associated with Poor Survival and an Immunosuppressive Tumor Microenvironment. Front. Immunol..

[33] Xu X., Bok I., Jasani N., Wang K., Chadourne M., Mecozzi N., Deng O., Welsh E.A., Kinose F., Rix U. (2024). PTEN Lipid Phosphatase Activity Suppresses Melanoma Formation by Opposing an AKT/mTOR/FRA1 Signaling Axis. Cancer Res..

[34] Grzymajlo K., El Hafny-Rahbi B., Kieda C. (2023). Tumour suppressor PTEN activity is differentially inducible by myo-inositol phosphates. J. Cell. Mol. Med..

[35] Parkman G.L., Xu X., Holmen S.L., Karreth F.A. (2025). The Roles of PTEN in Melanoma Suppression. Pigment. Cell Melanoma Res..

[36] O’Bryan J.P., Frye R.A., Cogswell P.C., Neubauer A., Kitch B., Prokop C., Espinosa R., Le Beau M.M., Earp H.S., Liu E.T. (1991). *axl*, a transforming gene isolated from primary human myeloid leukemia cells, encodes a novel receptor tyrosine kinase. Mol. Cell. Biol..

[37] Wessely A., Steeb T., Berking C., Heppt M.V. (2021). How Neural Crest Transcription Factors Contribute to Melanoma Heterogeneity, Cellular Plasticity, and Treatment Resistance. Int. J. Mol. Sci..

[38] Sensi M., Catani M., Castellano G., Nicolini G., Alciato F., Tragni G., De Santis G., Bersani I., Avanzi G., Tomassetti A. (2011). Human cutaneous melanomas lacking MITF and melanocyte differentiation antigens express a functional Axl receptor kinase. J. Investig. Dermatol..

[39] Strub T., Giuliano S., Ye T., Bonet C., Keime C., Kobi D., Le Gras S., Cormont M., Ballotti R., Bertolotto C. (2011). Essential role of microphthalmia transcription factor for DNA replication, mitosis and genomic stability in melanoma. Oncogene.

[40] Carreira S., Goodall J., Denat L., Rodriguez M., Nuciforo P., Hoek K.S., Testori A., Larue L., Goding C.R. (2006). Mitf regulation of Dia1 controls melanoma proliferation and invasiveness. Genes Dev..

[41] Nieto M.A., Huang R.Y., Jackson R.A., Thiery J.P. (2016). EMT: 2016. Cell.

[42] Zipser M.C., Eichhoff O.M., Widmer D.S., Schlegel N.C., Schoenewolf N.L., Stuart D., Liu W., Gardner H., Smith P.D., Nuciforo P. (2011). A proliferative melanoma cell phenotype is responsive to RAF/MEK inhibition independent of BRAF mutation status. Pigment. Cell Melanoma Res..

[43] Pedri D., Karras P., Landeloos E., Marine J.C., Rambow F. (2022). Epithelial-to-mesenchymal-like transition events in melanoma. FEBS J..

[44] Tsoi J., Robert L., Paraiso K., Galvan C., Sheu K.M., Lay J., Wong D.J.L., Atefi M., Shirazi R., Wang X. (2018). Multi-stage Differentiation Defines Melanoma Subtypes with Differential Vulnerability to Drug-Induced Iron-Dependent Oxidative Stress. Cancer Cell.

[45] Oliveira M., Lemos C.S., Brazão M.L.S., Rodrigues A.L.A., do Nascimento E.D.C., Cardoso E.M.P., Arantes M.F.F., de Melo F.F. (2026). Immune landscape of melanoma: Tumor microenvironment, resistance mechanisms, and predictive biomarkers. World J. Clin. Oncol..

[46] Zhang H., Yue X., Chen Z., Liu C., Wu W., Zhang N., Liu Z., Yang L., Jiang Q., Cheng Q. (2023). Define cancer-associated fibroblasts (CAFs) in the tumor microenvironment: New opportunities in cancer immunotherapy and advances in clinical trials. Mol. Cancer.

[47] Sikorski H., Żmijewski M.A., Piotrowska A. (2025). Tumor Microenvironment in Melanoma-Characteristic and Clinical Implications. Int. J. Mol. Sci..

[48] Anderson N.M., Simon M.C. (2020). The tumor microenvironment. Curr. Biol..

[49] Di Gennaro P., Gerlini G., Caporale R., Sestini S., Brandani P., Urso C., Pimpinelli N., Borgognoni L. (2018). T regulatory cells mediate immunosuppresion by adenosine in peripheral blood, sentinel lymph node and TILs from melanoma patients. Cancer Lett..

[50] Di Gennaro P., Gerlini G., Urso C., Sestini S., Brandani P., Pimpinelli N., Borgognoni L. (2016). CD4^+^FOXP3^+^ T regulatory cells decrease and CD3^+^CD8^+^ T cells recruitment in TILs from melanoma metastases after electrochemotherapy. Clin. Exp. Metastasis.

[51] Christofides A., Strauss L., Yeo A., Cao C., Charest A., Boussiotis V.A. (2022). The complex role of tumor-infiltrating macrophages. Nat. Immunol..

[52] Basak U., Sarkar T., Mukherjee S., Chakraborty S., Dutta A., Dutta S., Nayak D., Kaushik S., Das T., Sa G. (2023). Tumor-associated macrophages: An effective player of the tumor microenvironment. Front. Immunol..

[53] Chen C., Zhou S., Yang X., Ren M., Qi Y., Mao Y., Yang C. (2024). In vitro study of cold atmospheric plasma-activated liquids inhibits malignant melanoma by affecting macrophage polarization through the ROS/JAK2/STAT1 pathway. Biomed. Pharmacother..

[54] Yin J., Forn-Cuní G., Surendran A.M., Lopes-Bastos B., Pouliopoulou N., Jager M.J., Le Dévédec S.E., Chen Q., Snaar-Jagalska B.E. (2024). Lactate secreted by glycolytic conjunctival melanoma cells attracts and polarizes macrophages to drive angiogenesis in zebrafish xenografts. Angiogenesis.

[55] Pizzurro G.A., Liu C., Bridges K., Alexander A.F., Huang A., Baskaran J.P., Ramseier J., Bosenberg M.W., Mak M., Miller-Jensen K. (2021). 3D Model of the Early Melanoma Microenvironment Captures Macrophage Transition into a Tumor-Promoting Phenotype. Cancers.

[56] Chen Y., Zhou J., Liu Z., Wu T., Li S., Zhang Y., Yin X., Yang G., Zhang G. (2023). Tumor cell-induced platelet aggregation accelerates hematogenous metastasis of malignant melanoma by triggering macrophage recruitment. J. Exp. Clin. Cancer Res..

[57] Romano V., Belviso I., Venuta A., Ruocco M.R., Masone S., Aliotta F., Fiume G., Montagnani S., Avagliano A., Arcucci A. (2021). Influence of Tumor Microenvironment and Fibroblast Population Plasticity on Melanoma Growth, Therapy Resistance and Immunoescape. Int. J. Mol. Sci..

[58] Avagliano A., Arcucci A. (2022). Insights into Melanoma Fibroblast Populations and Therapeutic Strategy Perspectives: Friends or Foes?. Curr. Med. Chem..

[59] Moretti L., Stalfort J., Barker T.H., Abebayehu D. (2022). The interplay of fibroblasts, the extracellular matrix, and inflammation in scar formation. J. Biol. Chem..

[60] Papaccio F., Kovacs D., Bellei B., Caputo S., Migliano E., Cota C., Picardo M. (2021). Profiling Cancer-Associated Fibroblasts in Melanoma. Int. J. Mol. Sci..

[61] Forsthuber A., Aschenbrenner B., Korosec A., Jacob T., Annusver K., Krajic N., Kholodniuk D., Frech S., Zhu S., Purkhauser K. (2024). Cancer-associated fibroblast subtypes modulate the tumor-immune microenvironment and are associated with skin cancer malignancy. Nat. Commun..

[62] Wenthe J., Naseri S., Hellström A.C., Moreno R., Ullenhag G., Alemany R., Lövgren T., Eriksson E., Loskog A. (2022). Immune priming using DC- and T cell-targeting gene therapy sensitizes both treated and distant B16 tumors to checkpoint inhibition. Mol. Ther. Oncolytics.

[63] Brocard A., Knol A.C., Bossard C., Denis M.G., Quéreux G., Saint-Jean M., Peuvrel L., Khammari A., Blancho G., Dantal J. (2017). Clinical, Genetic and Innate Immunity Characteristics of Melanoma in Organ Transplant Recipients. Acta Derm. Venereol..

[64] Rubel F., Kern J.S., Technau-Hafsi K., Uhrich S., Thoma K., Häcker G., von Bubnoff N., Meiss F., von Bubnoff D. (2018). Indoleamine 2,3-Dioxygenase Expression in Primary Cutaneous Melanoma Correlates with Breslow Thickness and Is of Significant Prognostic Value for Progression-Free Survival. J. Investig. Dermatol..

[65] Najem A., Soumoy L., Sabbah M., Krayem M., Awada A., Journe F., Ghanem G.E. (2022). Understanding Molecular Mechanisms of Phenotype Switching and Crosstalk with TME to Reveal New Vulnerabilities of Melanoma. Cells.

[66] Dixit T., Aswini A., Nikam H., Vaidya A., Ravindran S. (2025). Emerging trends in synthesis, characterization, and mechanism of action of antibody-drug and antibody-nanoparticle conjugates. Discov. Nano.

[67] Perrot C.Y., Javelaud D., Mauviel A. (2013). Insights into the Transforming Growth Factor-β Signaling Pathway in Cutaneous Melanoma. Ann. Dermatol..

[68] Liu X., Ding Q., Zhang H., Zhang X., Chen Q., Weng S. (2025). The CD39-CD73-adenosine axis: Master regulator of immune evasion and therapeutic target in pancreatic ductal adenocarcinoma. Biochim. Biophys. Acta Rev. Cancer.

[69] Johnson D.B., Menzies A.M., Zimmer L., Eroglu Z., Ye F., Zhao S., Rizos H., Sucker A., Scolyer R.A., Gutzmer R. (2015). Acquired BRAF inhibitor resistance: A multicenter meta-analysis of the spectrum and frequencies, clinical behaviour, and phenotypic associations of resistance mechanisms. Eur. J. Cancer.

[70] Long G.V., Fung C., Menzies A.M., Pupo G.M., Carlino M.S., Hyman J., Shahheydari H., Tembe V., Thompson J.F., Saw R.P. (2014). Increased MAPK reactivation in early resistance to dabrafenib/trametinib combination therapy of BRAF-mutant metastatic melanoma. Nat. Commun..

[71] Ziogas D.C., Theocharopoulos C., Koutouratsas T., Haanen J., Gogas H. (2023). Mechanisms of resistance to immune checkpoint inhibitors in melanoma: What we have to overcome?. Cancer Treat. Rev..

[72] Meric-Bernstam F., Larkin J., Tabernero J., Bonini C. (2021). Enhancing anti-tumour efficacy with immunotherapy combinations. Lancet.

[73] Lauss M., Phung B., Borch T.H., Harbst K., Kaminska K., Ebbesson A., Hedenfalk I., Yuan J., Nielsen K., Ingvar C. (2024). Molecular patterns of resistance to immune checkpoint blockade in melanoma. Nat. Commun..

[74] Turner L.M., Terhaar H., Jiminez V., Anderson B.J., Grant E., Yusuf N. (2025). Tumor Microenvironmental Dynamics in Shaping Resistance to Therapeutic Interventions in Melanoma: A Narrative Review. Pharmaceuticals.

[75] Villanueva J., Vultur A., Lee J.T., Somasundaram R., Fukunaga-Kalabis M., Cipolla A.K., Wubbenhorst B., Xu X., Gimotty P.A., Kee D. (2010). Acquired resistance to BRAF inhibitors mediated by a RAF kinase switch in melanoma can be overcome by cotargeting MEK and IGF-1R/PI3K. Cancer Cell.

[76] Wang B., Zhang W., Zhang G., Kwong L., Lu H., Tan J., Sadek N., Xiao M., Zhang J., Labrie M. (2021). Targeting mTOR signaling overcomes acquired resistance to combined BRAF and MEK inhibition in BRAF-mutant melanoma. Oncogene.

[77] Chamcheu J.C., Roy T., Uddin M.B., Banang-Mbeumi S., Chamcheu R.N., Walker A.L., Liu Y.Y., Huang S. (2019). Role and Therapeutic Targeting of the PI3K/Akt/mTOR Signaling Pathway in Skin Cancer: A Review of Current Status and Future Trends on Natural and Synthetic Agents Therapy. Cells.

[78] Mendoza M.C., Er E.E., Blenis J. (2011). The Ras-ERK and PI3K-mTOR pathways: Cross-talk and compensation. Trends Biochem. Sci..

[79] Tolcher A.W., Peng W., Calvo E. (2018). Rational Approaches for Combination Therapy Strategies Targeting the MAP Kinase Pathway in Solid Tumors. Mol. Cancer Ther..

[80] Vido M.J., Le K., Hartsough E.J., Aplin A.E. (2018). BRAF Splice Variant Resistance to RAF Inhibitor Requires Enhanced MEK Association. Cell Rep..

[81] Pavelescu L.A., Enache R.M., Roşu O.A., Profir M., Creţoiu S.M., Gaspar B.S. (2024). Predictive Biomarkers and Resistance Mechanisms of Checkpoint Inhibitors in Malignant Solid Tumors. Int. J. Mol. Sci..

[82] Sivan A., Corrales L., Hubert N., Williams J.B., Aquino-Michaels K., Earley Z.M., Benyamin F.W., Lei Y.M., Jabri B., Alegre M.L. (2015). Commensal Bifidobacterium promotes antitumor immunity and facilitates anti-PD-L1 efficacy. Science.

[83] Indini A., Massi D., Pirro M., Roila F., Grossi F., Sahebkar A., Glodde N., Bald T., Mandalà M. (2022). Targeting inflamed and non-inflamed melanomas: Biological background and clinical challenges. Semin. Cancer Biol..

[84] Tutuka C.S.A., Andrews M.C., Mariadason J.M., Ioannidis P., Hudson C., Cebon J., Behren A. (2017). PLX8394, a new generation BRAF inhibitor, selectively inhibits BRAF in colonic adenocarcinoma cells and prevents paradoxical MAPK pathway activation. Mol. Cancer.

[85] Degirmenci U., Yap J., Sim Y.R.M., Qin S., Hu J. (2021). Drug resistance in targeted cancer therapies with RAF inhibitors. Cancer Drug Resist..

[86] Yaeger R., Corcoran R.B. (2019). Targeting Alterations in the RAF-MEK Pathway. Cancer Discov..

[87] Vanni I., Tanda E.T., Spagnolo F., Andreotti V., Bruno W., Ghiorzo P. (2020). The Current State of Molecular Testing in the BRAF-Mutated Melanoma Landscape. Front. Mol. Biosci..

[88] Desai J., Gan H., Barrow C., Jameson M., Atkinson V., Haydon A., Millward M., Begbie S., Brown M., Markman B. (2020). Phase I, Open-Label, Dose-Escalation/Dose-Expansion Study of Lifirafenib (BGB-283), an RAF Family Kinase Inhibitor, in Patients with Solid Tumors. J. Clin. Oncol..

[89] Mechahougui H., Gutmans J., Gouasmi R., Smekens L., Friedlaender A. (2025). BRAF Targeting Across Solid Tumors: Molecular Aspects and Clinical Applications. Int. J. Mol. Sci..

[90] Khaddour K., Kurn H., Patel P., Zito P.M. (2026). Vemurafenib. StatPearls.

[91] Germann U.A., Furey B.F., Markland W., Hoover R.R., Aronov A.M., Roix J.J., Hale M., Boucher D.M., Sorrell D.A., Martinez-Botella G. (2017). Targeting the MAPK Signaling Pathway in Cancer: Promising Preclinical Activity with the Novel Selective ERK1/2 Inhibitor BVD-523 (Ulixertinib). Mol. Cancer Ther..

[92] Sullivan R.J., Infante J.R., Janku F., Wong D.J.L., Sosman J.A., Keedy V., Patel M.R., Shapiro G.I., Mier J.W., Tolcher A.W. (2018). First-in-Class ERK1/2 Inhibitor Ulixertinib (BVD-523) in Patients with MAPK Mutant Advanced Solid Tumors: Results of a Phase I Dose-Escalation and Expansion Study. Cancer Discov..

[93] Buchbinder E.I., Cohen J.V., Tarantino G., Lian C.G., Liu D., Haq R., Hodi F.S., Lawrence D.P., Giobbie-Hurder A., Knoerzer D. (2024). A Phase II Study of ERK Inhibition by Ulixertinib (BVD-523) in Metastatic Uveal Melanoma. Cancer Res. Commun..

[94] Müller S.M., Jücker M. (2024). The Functional Roles of the Src Homology 2 Domain-Containing Inositol 5-Phosphatases SHIP1 and SHIP2 in the Pathogenesis of Human Diseases. Int. J. Mol. Sci..

[95] Molina J.R., Sun Y., Protopopova M., Gera S., Bandi M., Bristow C., McAfoos T., Morlacchi P., Ackroyd J., Agip A.A. (2018). An inhibitor of oxidative phosphorylation exploits cancer vulnerability. Nat. Med..

[96] Haq R., Shoag J., Andreu-Perez P., Yokoyama S., Edelman H., Rowe G.C., Frederick D.T., Hurley A.D., Nellore A., Kung A.L. (2013). Oncogenic BRAF regulates oxidative metabolism via PGC1α and MITF. Cancer Cell.

[97] Tsuji A., Akao T., Masuya T., Murai M., Miyoshi H. (2020). IACS-010759, a potent inhibitor of glycolysis-deficient hypoxic tumor cells, inhibits mitochondrial respiratory complex I through a unique mechanism. J. Biol. Chem..

[98] Yap T.A., Daver N., Mahendra M., Zhang J., Kamiya-Matsuoka C., Meric-Bernstam F., Kantarjian H.M., Ravandi F., Collins M.E., Francesco M.E.D. (2023). Complex I inhibitor of oxidative phosphorylation in advanced solid tumors and acute myeloid leukemia: Phase I trials. Nat. Med..

[99] Baenke F., Chaneton B., Smith M., Van Den Broek N., Hogan K., Tang H., Viros A., Martin M., Galbraith L., Girotti M.R. (2016). Resistance to BRAF inhibitors induces glutamine dependency in melanoma cells. Mol. Oncol..

[100] Nassief G., Anaeme A., Moussa K., Mansour A.N., Ansstas G. (2024). Recent Advancements in Cell-Based Therapies in Melanoma. Int. J. Mol. Sci..

[101] Knight A., Karapetyan L., Kirkwood J.M. (2023). Immunotherapy in Melanoma: Recent Advances and Future Directions. Cancers.

[102] Wolchok J.D., Chiarion-Sileni V., Rutkowski P., Cowey C.L., Schadendorf D., Wagstaff J., Queirolo P., Dummer R., Butler M.O., Hill A.G. (2025). Final, 10-Year Outcomes with Nivolumab plus Ipilimumab in Advanced Melanoma. N. Engl. J. Med..

[103] Tang W., Chen J., Ji T., Cong X. (2023). TIGIT, a novel immune checkpoint therapy for melanoma. Cell Death Dis..

[104] Buchanan T., Amouzegar A., Luke J.J. (2021). Next-Generation Immunotherapy Approaches in Melanoma. Curr. Oncol. Rep..

[105] Lu C., Tan Y. (2024). Promising immunotherapy targets: TIM3, LAG3, and TIGIT joined the party. Mol. Ther. Oncol..

[106] Tawbi H.A., Hodi F.S., Lipson E.J., Schadendorf D., Ascierto P.A., Matamala L., Castillo Gutiérrez E., Rutkowski P., Gogas H., Lao C.D. (2025). Three-Year Overall Survival with Nivolumab Plus Relatlimab in Advanced Melanoma from RELATIVITY-047. J. Clin. Oncol..

[107] Lee W.J., Lee Y.J., Choi M.E., Yun K.A., Won C.H., Lee M.W., Choi J.H., Chang S.E. (2019). Expression of lymphocyte-activating gene 3 and T-cell immunoreceptor with immunoglobulin and ITIM domains in cutaneous melanoma and their correlation with programmed cell death 1 expression in tumor-infiltrating lymphocytes. J. Am. Acad. Dermatol..

[108] Diab A., Gogas H., Sandhu S., Long G.V., Ascierto P.A., Larkin J., Sznol M., Franke F., Ciuleanu T.E., Pereira C. (2023). Bempegaldesleukin Plus Nivolumab in Untreated Advanced Melanoma: The Open-Label, Phase III PIVOT IO 001 Trial Results. J. Clin. Oncol..

[109] Yang Y., Lundqvist A. (2020). Immunomodulatory Effects of IL-2 and IL-15; Implications for Cancer Immunotherapy. Cancers.

[110] Montorfani J., Hatterer E., Chatel L., Lesnier A., Viandier A., Daubeuf B., Nouveau L., Malinge P., Calloud S., Masternak K. (2025). Selective activation of interleukin-2/interleukin-15 receptor signaling in tumor microenvironment using paired bispecific antibodies. J. Immunother. Cancer.

[111] Mortensen M.R., Mock J., Bertolini M., Stringhini M., Catalano M., Neri D. (2020). Targeting an engineered cytokine with interleukin-2 and interleukin-15 activity to the neovasculature of solid tumors. Oncotarget.

[112] Glitza I.C., Goff S.L., Ross M., Margolin K. (2020). And Now for Something Completely Different: Immunotherapy Beyond Checkpoints in Melanoma. Am. Soc. Clin. Oncol. Educ. Book.

[113] Tsai K.K., Komanduri K.V. (2025). Tumor-Infiltrating Lymphocyte Therapy for the Treatment of Metastatic Melanoma. Am. J. Clin. Dermatol..

[114] Medina T., Chesney J.A., Kluger H.M., Hamid O., Whitman E.D., Cusnir M., Thomas S.S., Wermke M., Domingo-Musibay E., Phan G.Q. (2025). Long-Term Efficacy and Safety of Lifileucel Tumor-Infiltrating Lymphocyte Cell Therapy in Patients with Advanced Melanoma: A 5-Year Analysis of the C-144-01 Study. J. Clin. Oncol..

[115] Rabbani S.A., El-Tanani M., El-Tanani Y., Kumar R., Sharma S., Khan M.A., Parvez S., Aljabali A.A.A., Matalka M.I., Rizzo M. (2025). Advances in Adoptive Cell Therapies in Cancer: From Mechanistic Breakthroughs to Clinical Frontiers and Overcoming Barriers. Med. Sci..

[116] Reinhorn D., Tak W.S., Haanen J., D’Angelo S.P., Fashoyin-Aje L.A. (2025). Autologous T-Cell Therapies in Solid Tumor Malignancies: Current Landscape and Future Opportunities. Am. Soc. Clin. Oncol. Educ. Book.

[117] Escobar G., Berger T.R., Maus M.V. (2025). CAR-T cells in solid tumors: Challenges and breakthroughs. Cell Rep. Med..

[118] Boti M.A., Diamantopoulos M.A., Scorilas A. (2025). RNA-Targeting Techniques: A Comparative Analysis of Modern Approaches for RNA Manipulation in Cancer Research and Therapeutics. Genes.

[119] Viola J.R., Rafael D.F., Wagner E., Besch R., Ogris M. (2013). Gene therapy for advanced melanoma: Selective targeting and therapeutic nucleic acids. J. Drug Deliv..

[120] Wang D., Jia W., Lian J., Zhang J., Ren X., Wang Z., Liu W., Zhou C., Xu J., Tian W. (2026). Identification of siRNA silencing TGFβ1 and VEGFR2 and utility of the combination in targeting immune modulation and angiogenesis in treating melanoma in vivo. NAR Cancer.

[121] Feichtenschlager V., Zheng Y.J., Ho W., Chen L., Callanan C., Chen C., Lee A., Ortiz J., Rappersberger K., Ortiz-Urda S. (2023). Deconstructing the role of MALAT1 in MAPK-signaling in melanoma: Insights from antisense oligonucleotide treatment. Oncotarget.

[122] Bidram M., Zhao Y., Shebardina N.G., Baldin A.V., Bazhin A.V., Ganjalikhany M.R., Zamyatnin A.A., Ganjalikhani-Hakemi M. (2021). mRNA-Based Cancer Vaccines: A Therapeutic Strategy for the Treatment of Melanoma Patients. Vaccines.

[123] Bafaloukos D., Gazouli I., Koutserimpas C., Samonis G. (2023). Evolution and Progress of mRNA Vaccines in the Treatment of Melanoma: Future Prospects. Vaccines.

[124] Weber J.S., Carlino M.S., Khattak A., Meniawy T., Ansstas G., Taylor M.H., Kim K.B., McKean M., Long G.V., Sullivan R.J. (2024). Individualised neoantigen therapy mRNA-4157 (V940) plus pembrolizumab versus pembrolizumab monotherapy in resected melanoma (KEYNOTE-942): A randomised, phase 2b study. Lancet.

[125] Sisk C.K., Turner L.M., Meraj S., Yusuf N. (2026). Advances in mRNA-Based Melanoma Vaccines: A Narrative Review of Lipid Nanoparticle and Dendritic Cell Delivery Platforms. Cells.

[126] Moazzam M., Zhang M., Hussain A., Yu X., Huang J., Huang Y. (2024). The landscape of nanoparticle-based siRNA delivery and therapeutic development. Mol. Ther..

[127] Paulo E.P.A., Cabral L.G.S., Poyet J.L., Maria D.A. (2026). Nanotechnology in Cutaneous Oncology: The Role of Liposomes in Targeted Melanoma Therapy. Molecules.

[128] Villares G.J., Zigler M., Wang H., Melnikova V.O., Wu H., Friedman R., Leslie M.C., Vivas-Mejia P.E., Lopez-Berestein G., Sood A.K. (2008). Targeting melanoma growth and metastasis with systemic delivery of liposome-incorporated protease-activated receptor-1 small interfering RNA. Cancer Res..

[129] Jia G., Jiang Y., Li X. (2024). Targeted drug conjugates in cancer therapy: Challenges and opportunities. Pharm. Sci. Adv..

[130] Gouda M.A., Subbiah V. (2022). Strategies for Mitigating Antibody-Drug Conjugate Related Adverse Events for Precision Therapy. Cancer J..

[131] Hoffmann R.M., Crescioli S., Mele S., Sachouli E., Cheung A., Chui C.K., Andriollo P., Jackson P.J.M., Lacy K.E., Spicer J.F. (2020). A Novel Antibody-Drug Conjugate (ADC) Delivering a DNA Mono-Alkylating Payload to Chondroitin Sulfate Proteoglycan (CSPG4)-Expressing Melanoma. Cancers.

[132] Goodman R., Johnson D.B. (2022). Antibody-Drug Conjugates for Melanoma and Other Skin Malignancies. Curr. Treat. Options Oncol..

[133] Lami I., Wiemer A.J. (2024). Antibody-Drug Conjugates in the Pipeline for Treatment of Melanoma: Target and Pharmacokinetic Considerations. Drugs R D.

[134] Kidwai N., Chen M., Postow M.A., Hassel J., Callahan M. (2024). Breaking the Mold: Trailblazing Melanoma Therapy Beyond Checkpoint Through Innovative Approaches. Am. Soc. Clin. Oncol. Educ. Book.

[135] Zhou M., Huang Z., Ma Z., Chen J., Lin S., Yang X., Gong Q., Braunstein Z., Wei Y., Rao X. (2025). The next frontier in antibody-drug conjugates: Challenges and opportunities in cancer and autoimmune therapy. Cancer Drug Resist..

[136] Wang R., Hu B., Pan Z., Mo C., Zhao X., Liu G., Hou P., Cui Q., Xu Z., Wang W. (2025). Antibody-Drug Conjugates (ADCs): Current and future biopharmaceuticals. J. Hematol. Oncol..

[137] Buyukgolcigezli I., Tenekeci A.K., Sahin I.H. (2025). Opportunities and Challenges in Antibody-Drug Conjugates for Cancer Therapy: A New Era for Cancer Treatment. Cancers.

[138] Khongorzul P., Ling C.J., Khan F.U., Ihsan A.U., Zhang J. (2020). Antibody-Drug Conjugates: A Comprehensive Review. Mol. Cancer Res..

[139] Khorramdelazad H., Pirsadeghi A., Amraei E., Amani A., Abbasnia M., Nematzade F., Shahabinejad E., Mahabadi R.N., Ghanizadeh S., Kouchakzade F.S. (2026). Hydrogel-based delivery of cytokines in cancer immunotherapy: Latest evidence and therapeutic outcomes. Biomed. Pharmacother..

[140] Zheng M., Huang J., Tong A., Yang H. (2019). Oncolytic Viruses for Cancer Therapy: Barriers and Recent Advances. Mol. Ther. Oncolytics.

[141] Mohammadzadeh V., Atapour-Mashhad H., Shahvali S., Salehi B., Shaban M., Shirzad M., Salahvarzi A., Mohammadi M. (2025). Hydrogels as advanced drug delivery platforms for cancer immunotherapy: Promising innovations and future outlook. J. Nanobiotechnol..

[142] Kim J., Archer P.A., Manspeaker M.P., Avecilla A.R.C., Pollack B.P., Thomas S.N. (2023). Sustained release hydrogel for durable locoregional chemoimmunotherapy for BRAF-mutated melanoma. J. Control. Release.

[143] Pacheco C., Baião A., Ding T., Cui W., Sarmento B. (2023). Recent advances in long-acting drug delivery systems for anticancer drug. Adv. Drug Deliv. Rev..

[144] Das S., Khuda-Bukhsh A.R. (2016). PLGA-loaded nanomedicines in melanoma treatment: Future prospect for efficient drug delivery. Indian J. Med. Res..

[145] Phoon Y.P., Wolfarth A.A., Funchain P., Ko J.S., Choi D., Dedkova E., Bateman D.D., Corvin J., Melenhorst J.J., Gastman B.R. (2025). NT-I7, a novel long-acting interleukin-7, promotes anti-PD-1 efficacy in an autologous humanized melanoma model. Sci. Rep..

[146] Li J., Mooney D.J. (2016). Designing hydrogels for controlled drug delivery. Nat. Rev. Mater..

[147] Ng T.S.C., Hu H., Kronister S., Lee C., Li R., Gerosa L., Stopka S.A., Burgenske D.M., Khurana I., Regan M.S. (2022). Overcoming differential tumor penetration of BRAF inhibitors using computationally guided combination therapy. Sci. Adv..

[148] Díaz E., Quezada V., Cifuentes J., Arias Morales N.Y., Reyes L.H., Muñoz-Camargo C., Cruz J.C. (2024). Enhanced Delivery and Potency of Chemotherapeutics in Melanoma Treatment via Magnetite Nanobioconjugates. ACS Omega.

[149] Wei W., Rosenkrans Z.T., Liu J., Huang G., Luo Q.Y., Cai W. (2020). ImmunoPET: Concept, Design, and Applications. Chem. Rev..

[150] Aide N., Hicks R.J., Le Tourneau C., Lheureux S., Fanti S., Lopci E. (2019). FDG PET/CT for assessing tumour response to immunotherapy: Report on the EANM symposium on immune modulation and recent review of the literature. Eur. J. Nucl. Med. Mol. Imaging.

[151] Sullivan R.J., Flaherty K. (2013). MAP kinase signaling and inhibition in melanoma. Oncogene.

[152] Benito-Jardón L., Díaz-Martínez M., Arellano-Sánchez N., Vaquero-Morales P., Esparís-Ogando A., Teixidó J. (2019). Resistance to MAPK Inhibitors in Melanoma Involves Activation of the IGF1R-MEK5-Erk5 Pathway. Cancer Res..

[153] Welsh S.J., Rizos H., Scolyer R.A., Long G.V. (2016). Resistance to combination BRAF and MEK inhibition in metastatic melanoma: Where to next?. Eur. J. Cancer.

[154] Pratt E.C., Isaac E., Stater E.P., Yang G., Ouerfelli O., Pillarsetty N., Grimm J. (2020). Synthesis of the PET Tracer (124)I-Trametinib for MAPK/ERK Kinase Distribution and Resistance Monitoring. J. Nucl. Med..

[155] Henry K.E., Dacek M.M., Dilling T.R., Caen J.D., Fox I.L., Evans M.J., Lewis J.S. (2019). A PET Imaging Strategy for Interrogating Target Engagement and Oncogene Status in Pancreatic Cancer. Clin. Cancer Res..

[156] Bensch F., van der Veen E.L., Lub-de Hooge M.N., Jorritsma-Smit A., Boellaard R., Kok I.C., Oosting S.F., Schröder C.P., Hiltermann T.J.N., van der Wekken A.J. (2018). ^89^Zr-atezolizumab imaging as a non-invasive approach to assess clinical response to PD-L1 blockade in cancer. Nat. Med..

[157] Pandit-Taskar N., Mauguen A., Frosina D., Jungbluth A., Busam K.J., Lyashchenko S., Schwartz J., Momtaz P., Warner A.B., Smithy J.W. (2025). Programmed death ligand-1 PET imaging in patients with melanoma: A pilot study. Melanoma Res..

[158] Tavaré R., Escuin-Ordinas H., Mok S., McCracken M.N., Zettlitz K.A., Salazar F.B., Witte O.N., Ribas A., Wu A.M. (2016). An Effective Immuno-PET Imaging Method to Monitor CD8-Dependent Responses to Immunotherapy. Cancer Res..

[159] Rashidian M., Keliher E.J., Bilate A.M., Duarte J.N., Wojtkiewicz G.R., Jacobsen J.T., Cragnolini J., Swee L.K., Victora G.D., Weissleder R. (2015). Noninvasive imaging of immune responses. Proc. Natl. Acad. Sci. USA.

[160] Farwell M.D., Gamache R.F., Babazada H., Hellmann M.D., Harding J.J., Korn R., Mascioni A., Le W., Wilson I., Gordon M.S. (2022). CD8-Targeted PET Imaging of Tumor-Infiltrating T Cells in Patients with Cancer: A Phase I First-in-Humans Study of ^89^Zr-Df-IAB22M2C, a Radiolabeled Anti-CD8 Minibody. J. Nucl. Med..

[161] Scaini M.C., Catoni C., Poggiana C., Pigozzo J., Piccin L., Leone K., Scarabello I., Facchinetti A., Menin C., Elefanti L. (2024). A multiparameter liquid biopsy approach allows to track melanoma dynamics and identify early treatment resistance. npj Precis. Oncol..

[162] Slusher N., Jones N., Nonaka T. (2024). Liquid biopsy for diagnostic and prognostic evaluation of melanoma. Front. Cell Dev. Biol..

[163] Schroeder C., Gatidis S., Kelemen O., Schütz L., Bonzheim I., Muyas F., Martus P., Admard J., Armeanu-Ebinger S., Gückel B. (2024). Tumour-informed liquid biopsies to monitor advanced melanoma patients under immune checkpoint inhibition. Nat. Commun..

[164] Di Nardo L., Del Regno L., Di Stefani A., Mannino M., Fossati B., Catapano S., Quattrini L., Pellegrini C., Cortellini A., Parisi A. (2023). The dynamics of circulating tumour DNA (ctDNA) during treatment reflects tumour response in advanced melanoma patients. Exp. Dermatol..

[165] Gianmarco M., Carolina P., Gregorio M., Michela V., Monica P., Claire G.G., Michele M., Giulia M., Roberta M., Cinzia A. (2025). Circulating tumor DNA monitoring in advanced mutated melanoma (LIQUID-MEL). J. Liq. Biopsy.

[166] Luan W., Lu X., Peng H., Shen X., Rao M., Ruan H. (2024). Exosomal miR-19a derived from melanoma cell promotes the vemurafenib resistance of malignant melanoma through directly targeting LRIG1 to reactivate AKT and MAPK pathway. Pathol. Res. Pract..

[167] Fatima S., Ma Y., Safrachi A., Haider S., Spring K.J., Vafaee F., Scott K.F., Roberts T.L., Becker T.M., de Souza P. (2022). Harnessing Liquid Biopsies to Guide Immune Checkpoint Inhibitor Therapy. Cancers.

[168] Robert C., Grob J.J., Stroyakovskiy D., Karaszewska B., Hauschild A., Levchenko E., Chiarion Sileni V., Schachter J., Garbe C., Bondarenko I. (2019). Five-Year Outcomes with Dabrafenib plus Trametinib in Metastatic Melanoma. N. Engl. J. Med..

[169] Weide B., Elsässer M., Büttner P., Pflugfelder A., Leiter U., Eigentler T.K., Bauer J., Witte M., Meier F., Garbe C. (2012). Serum markers lactate dehydrogenase and S100B predict independently disease outcome in melanoma patients with distant metastasis. Br. J. Cancer.

[170] Andhari S., Farmaha J., Vashisht A., Vashisht V., Woodall J., Mondal A.K., Jones K., Pandita A., Shafi G., Uttarwar M. (2026). Real-Time Therapy Response Monitoring Using Surface Biomarkers on Circulating Tumor Cells. Cancers.

[171] Yadav P., Rajendrasozhan S., Lajimi R.H., Patel R.R., Heymann D., Prasad N.R. (2025). Circulating tumor cell markers for early detection and drug resistance assessment through liquid biopsy. Front. Oncol..

[172] Kloten V., Lampignano R., Krahn T., Schlange T. (2019). Circulating Tumor Cell PD-L1 Expression as Biomarker for Therapeutic Efficacy of Immune Checkpoint Inhibition in NSCLC. Cells.

[173] Jiang Z., He J., Zhang B., Wang L., Long C., Zhao B., Yang Y., Du L., Luo W., Hu J. (2023). A Potential “Anti-Warburg Effect” in Circulating Tumor Cell-mediated Metastatic Progression?. Aging Dis..

[174] Jeong Y., Du R., Zhu X., Yin S., Wang J., Cui H., Cao W., Lowenstein C.J. (2014). Histone deacetylase isoforms regulate innate immune responses by deacetylating mitogen-activated protein kinase phosphatase-1. J. Leukoc. Biol..

[175] Hugo W., Zaretsky J.M., Sun L., Song C., Moreno B.H., Hu-Lieskovan S., Berent-Maoz B., Pang J., Chmielowski B., Cherry G. (2016). Genomic and Transcriptomic Features of Response to Anti-PD-1 Therapy in Metastatic Melanoma. Cell.

[176] Falletta P., Sanchez-Del-Campo L., Chauhan J., Effern M., Kenyon A., Kershaw C.J., Siddaway R., Lisle R., Freter R., Daniels M.J. (2017). Translation reprogramming is an evolutionarily conserved driver of phenotypic plasticity and therapeutic resistance in melanoma. Genes Dev..

[177] Weinstein D., Leininger J., Hamby C., Safai B. (2014). Diagnostic and prognostic biomarkers in melanoma. J. Clin. Aesthet. Dermatol..

[178] Ascierto P.A., Stroyakovskiy D., Gogas H., Robert C., Lewis K., Protsenko S., Pereira R.P., Eigentler T., Rutkowski P., Demidov L. (2023). Overall survival with first-line atezolizumab in combination with vemurafenib and cobimetinib in BRAF(V600) mutation-positive advanced melanoma (IMspire150): Second interim analysis of a multicentre, randomised, phase 3 study. Lancet Oncol..

[179] Atkins M.B., Lee S.J., Chmielowski B., Tarhini A.A., Cohen G.I., Truong T.G., Moon H.H., Davar D., O’Rourke M., Stephenson J.J. (2023). Combination Dabrafenib and Trametinib Versus Combination Nivolumab and Ipilimumab for Patients with Advanced BRAF-Mutant Melanoma: The DREAMseq Trial-ECOG-ACRIN EA6134. J. Clin. Oncol..

[180] Ascierto P.A., Casula M., Bulgarelli J., Pisano M., Piccinini C., Piccin L., Cossu A., Mandalà M., Ferrucci P.F., Guidoboni M. (2024). Sequential immunotherapy and targeted therapy for metastatic BRAF V600 mutated melanoma: 4-year survival and biomarkers evaluation from the phase II SECOMBIT trial. Nat. Commun..

[181] Amaral T., Ottaviano M., Arance A., Blank C., Chiarion-Sileni V., Donia M., Dummer R., Garbe C., Gershenwald J.E., Gogas H. (2025). Cutaneous melanoma: ESMO Clinical Practice Guideline for diagnosis, treatment and follow-up. Ann. Oncol..

[182] Liu Y., Altreuter J., Bodapati S., Cristea S., Wong C.J., Wu C.J., Michor F. (2024). Predicting patient outcomes after treatment with immune checkpoint blockade: A review of biomarkers derived from diverse data modalities. Cell Genom..

[183] Yin Y., Zhang T., Wang Z. (2025). An attention-based mRNA transformer network for accurate prediction of melanoma response to immune checkpoint inhibitors. Sci. Rep..

[184] West J., Adler F., Gallaher J., Strobl M., Brady-Nicholls R., Brown J., Roberson-Tessi M., Kim E., Noble R., Viossat Y. (2023). A survey of open questions in adaptive therapy: Bridging mathematics and clinical translation. eLife.

[185] Prelaj A., Miskovic V., Zanitti M., Trovo F., Genova C., Viscardi G., Rebuzzi S.E., Mazzeo L., Provenzano L., Kosta S. (2024). Artificial intelligence for predictive biomarker discovery in immuno-oncology: A systematic review. Ann. Oncol..

